# 2D Materials for Efficient Photodetection: Overview, Mechanisms, Performance and UV-IR Range Applications

**DOI:** 10.3389/fchem.2022.905404

**Published:** 2022-05-20

**Authors:** Maria Malik, Muhammad Aamir Iqbal, Jeong Ryeol Choi, Phuong V. Pham

**Affiliations:** ^1^ Centre of Excellence in Solid State Physics, University of the Punjab, Lahore, Pakistan; ^2^ School of Materials Science and Engineering, Zhejiang University, Hangzhou, China; ^3^ Department of Nanoengineering, Kyonggi University, Suwon, Korea; ^4^ Hangzhou Global Scientific and Technological Innovation Center, School of Micro-Nano Electronics, Zhejiang University, Hangzhou, China

**Keywords:** 2D materials, mechanisms, graphene, TMDC, black phosphorus, heterostructures, performance factors, UV-IR photodetectors

## Abstract

Two-dimensional (2D) materials have been widely used in photodetectors owing to their diverse advantages in device fabrication and manipulation, such as integration flexibility, availability of optical operation through an ultrabroad wavelength band, fulfilling of photonic demands at low cost, and applicability in photodetection with high-performance. Recently, transition metal dichalcogenides (TMDCs), black phosphorus (BP), III-V materials, heterostructure materials, and graphene have emerged at the forefront as intriguing basics for optoelectronic applications in the field of photodetection. The versatility of photonic systems composed of these materials enables their wide range of applications, including facilitation of chemical reactions, speeding-up of responses, and ultrasensitive light detection in the ultraviolet (UV), visible, mid-infrared (MIR), and far-infrared (FIR) ranges. This review provides an overview, evaluation, recent advancements as well as a description of the innovations of the past few years for state-of-the-art photodetectors based on two-dimensional materials in the wavelength range from UV to IR, and on the combinations of different two-dimensional crystals with other nanomaterials that are appealing for a variety of photonic applications. The device setup, materials synthesis, operating methods, and performance metrics for currently utilized photodetectors, along with device performance enhancement factors, are summarized.

## 1 Introduction

Many photonic devices that we use on a daily basis rely on the conversion of light into electrical signals. Such devices are related to video imaging, biomedical imaging, cyber security, gas sensing, optical communications, motion tracking, and so on. Whereas the development of devices that have new photonic functions, as well as their large-scale production, is highly necessary, and such a development mainly depends on technological advances in the processing of elevated materials. Photodetection platforms with improved performance in connection with efficiency, speed, transparency, wavelength range, flexibility, and integrability are becoming more and more important as their application domains are diversified on a large scale. Two-dimensional materials are multilayered materials where the atoms from distinct layers interact *via* van-der-Waal interactions, and the constituent atoms in such interactions are held together by chemical bonds (Ferrari et al., 2015). Due to their exceptional features, 2D materials such as graphene, transition metal dichalcogenides, and black phosphorus have emerged as the most promising semiconductor materials in the fabrication of optoelectronic devices. Photodetectors (PDs) based on various 2D materials can be designed and fabricated considering bandgaps that are layer-dependent and adequately sized. Due to the unique properties of 2D materials, it is possible to generate diverse novel physical phenomena by stacking numerous 2D materials together (Coleman et al., 2011; Cheng et al., 2019).

**FIGURE 1:**
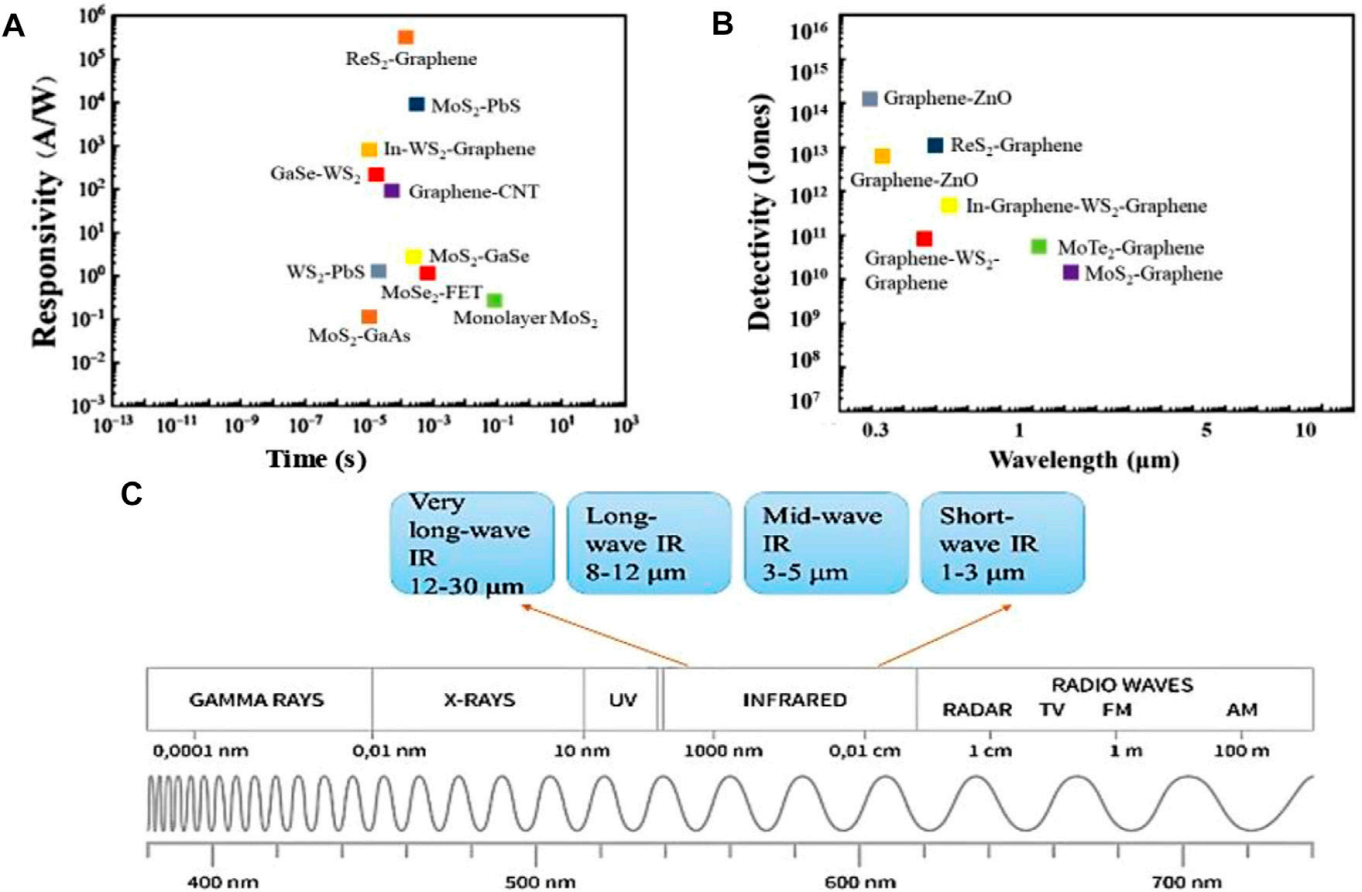
Performance comparison of various 2D materials-based photodetectors, **(A)** Photoresponsivity of conventional materials and their heterostructures for IR photodetection, **(B)** Detectivity of 2D materials and their heterostructures for IR photodetection, and **(C)** Electromagnetic spectrum along with targeted ranges as summarized in this study.

The 2D family of materials has grown significantly in recent years and now includes 2D materials of semi-metals (Liu et al., 2020), semiconductors (Chaves et al., 2020), and even insulators (Illarionov et al., 2020). Usually, photodetectors are indispensable components in numerous optoelectronic applications (Koppens et al., 2014; Long et al., 2019; Chen et al., 2020; Vicarelli et al., 2012). A distinct advantage of photodetectors fabricated with 2D materials is their ability to effectively detect the ultra-broad spectral bands ranging from visible light to infrared (IR) radiation (Long et al., 2019). Furthermore, 2D materials can be combined with a variety of material platforms, such as silicon and silicon compounds (Akinwande et al., 2019), III–V materials (Cao et al., 2020), and flexible materials (Chen et al., 2020; Deng et al., 2018a). A notable characteristic of these materials and compounds is that they do not have dangling bonds on their surface, which results in a minimized response of dark current, enabling researchers to employ them as efficient devices (Guan et al., 2021), as illustrated in Figure 1. Other intriguing properties of them include a wide range of bandgap, single-layered structure, efficient light-matter interactions, and heterostructure formations.

Graphene is a 2D substance that is comprised of single-layered carbon atoms organized in a hexagonal pattern. It has been discovered over the years that graphene retains numerous appealing electrical, mechanical, optical, and thermal properties (Novoselov et al., 2012; Iqbal et al., 2021a). Due to its semi-metallic character and zero bandgap property, graphene interacts with light across a broad spectrum of wavelengths, from terahertz to ultraviolet. As a result, it could be used in photodetectors with a broad-spectrum range. Graphene’s absorption coefficient is limited by its atomically thin nature, resulting in 2.3% absorption of the incident illumination beam. However, for optimal photocurrent generation and photodetector functioning, an enhanced absorption coefficient together with a higher lifespan of the photoexcited carriers is preferred (Li et al., 2014; Sun and Chang, 2014). To complement graphene’s low absorption coefficient, TMDCs have been considerably regarded in the last decade for optoelectronic applications. The advantage of TMDCs over graphene for wide-range photodetection is their semiconducting and varied bandgap nature. Despite the fact that the 2D-materials field is still in its early stages, the initial findings of optoelectronic devices depending on them are quite exciting. Over the last decade, the distinctive capabilities of 2D materials have also driven a huge volume of computational (Iqbal et al., 2021b) and experimental studies for their optoelectronic applications, and this has led to an extensive exploration of potential 2D materials.

Various III-V van-der-Waal multilayer-structure materials, including GaS (Hu et al., 2013), GaSe (Lei et al., 2013), and In_2_Se_3_ (Lin et al., 2013), are being explored as layered photodetector materials in addition to traditional 2D materials. Multi-layered GaSe has a good UV illumination response with low dark currents and high quantum efficiency. This is a natural consequence of its wide bandgap that increases from 1.8 to 3.2 eV as the number of layers is reduced. This consequence is caused by Se atoms’ pz-like orbitals that suppress interlayer contacts (Lei et al., 2013). Multi-layered InSe, besides, has a shorter indirect bandgap ranging from 1.2 to 1.4 eV, and has a relatively close overlap to the visible range of electromagnetic spectra (Lin et al., 2013). All of the aforementioned two-dimensional materials have applications in optoelectronics with a broadband detection range from visible to far-IR (Hu et al., 2013; Lei et al., 2013; Lin et al., 2013).

Most conventional photodetectors are available for achieving ultrasensitive detection, but only at low temperatures. The requirement for cryogenic protocols in the synthesis of them makes it difficult to attain a complex device structure along this line. However, the device preparation technique can be simplified if we use 2D materials. Furthermore, its high mobility and high light-matter interactions can enhance the photodetectors’ sensitivity and speed. In this review, we have summarized the detection mechanisms of the photodetectors along with their performance parameters and have explored the potential of 2D materials for ultra-sensitive photodetection for targeted wavelength spectral ranges.

## 2 Photodetection Mechanisms and Key Performance Parameters

The fundamental photonic effects, device configurations, and working mechanisms along with key performance parameters of 2D materials-based photodetectors are summarized in this section, wherein we have focused on photovoltaic effect (Xiao et al., 2015), photo-thermoelectric effect (Wang et al., 2020), photoconductive effect (Long et al., 2019), photogating effect (Koppens et al., 2014), bolometric effect (Kasirga, 2020), and plasma wave assisted mechanisms (Rupper et al., 2015), along with responsivity, detection, quantum efficiency, phot-response time, and photo-conductive gain.

### 2.1 Photodetection Mechanisms

#### 2.1.1 Photovoltaic Effect

**FIGURE 2:**
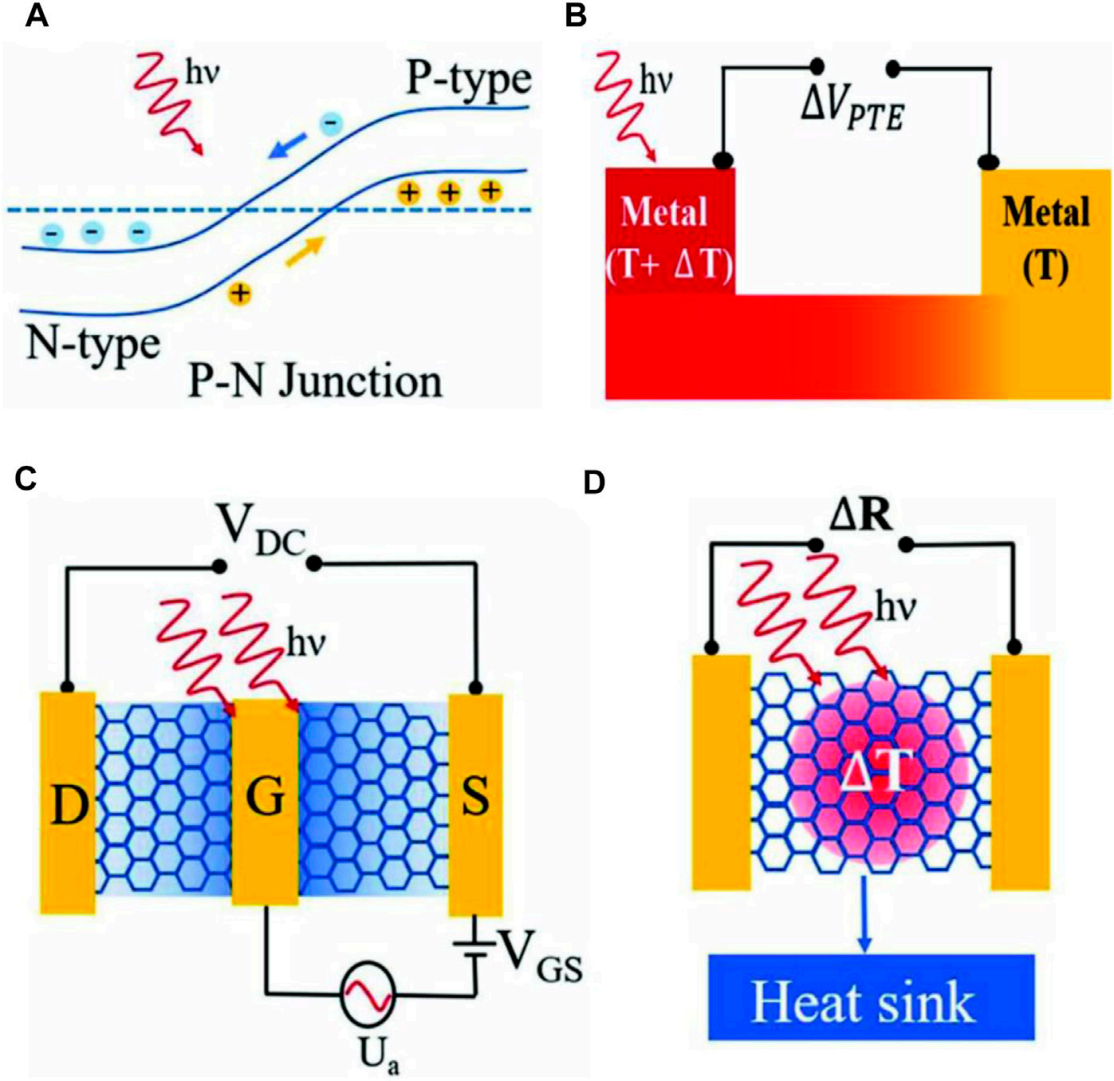
A schematic representation of photodetection mechanisms, **(A)** Photovoltaic effect (Qiu and Huang, 2021), **(B)** Photo-thermoelectric effect (Qiu and Huang, 2021), **(C)** Bolometric effect (Qiu and Huang, 2021), and **(D)** Plasma wave assisted mechanism (Qiu and Huang, 2021); Reproduced with permission from (Qiu and Huang, 2021).

The photovoltaic effect is attributed to the photogenerated charge carrier’s separation by electric field in the junction between n-type and p-type doped regions (Xiao et al., 2015). This effect can also be generated through an external electric field that can be produced by applying a bias voltage between source and drain. However, this procedure is not as favorable as it generates the dark current, as can be confirmed from Figure 2A (Qiu and Huang, 2021). The intrinsic electric field can be generated either by local chemical doping using split gates or through the difference of work function between the active material and metal contacts (Long et al., 2019). The generated photocurrent flows in the direction of the electric field and does not depend on the doping level. Thus, the change of the sign in the current flow is observed from pn to np and vice versa. The conversion of a high-energy electron-hole pair into many low-energy pairs, which is also known as carrier multiplication, occurs as a result of e-e scattering, resulting in effective photodetection (Frisenda et al., 2018).

#### 2.1.2 Photo-Thermoelectric Effect

The photo-thermoelectric effect (Wang et al., 2020) is the indirect conversion of light into electrical signals caused by variations in light absorption factors in different regions. The change of electron thermal motion arisen due to this effect in an active region creates a temperature gradient within the device, as shown in Figure 2B (Qiu and Huang, 2021). These thermal movements increase the temperature of the active material according to the Seebeck effect (Adhikari, 2017), which leads to a change of its electronic properties and so modifies the material’s potential difference. The Seebeck effect can be explained by the emergence of photo-thermoelectric voltage between different regions of which temperature is not the same. Photo-thermoelectric voltage is governed by the equation V_PTE_=(S_2_–S_1_)∆T, where ∆T is the temperature difference at two regions, whereas S_1_ and S_2_ are thermoelectric coefficients of the active material. The scale of the thermoelectric coefficient is closely related to the conductance in a region (Adhikari, 2017).

#### 2.1.3 Photoconductive Effect

**FIGURE 3:**
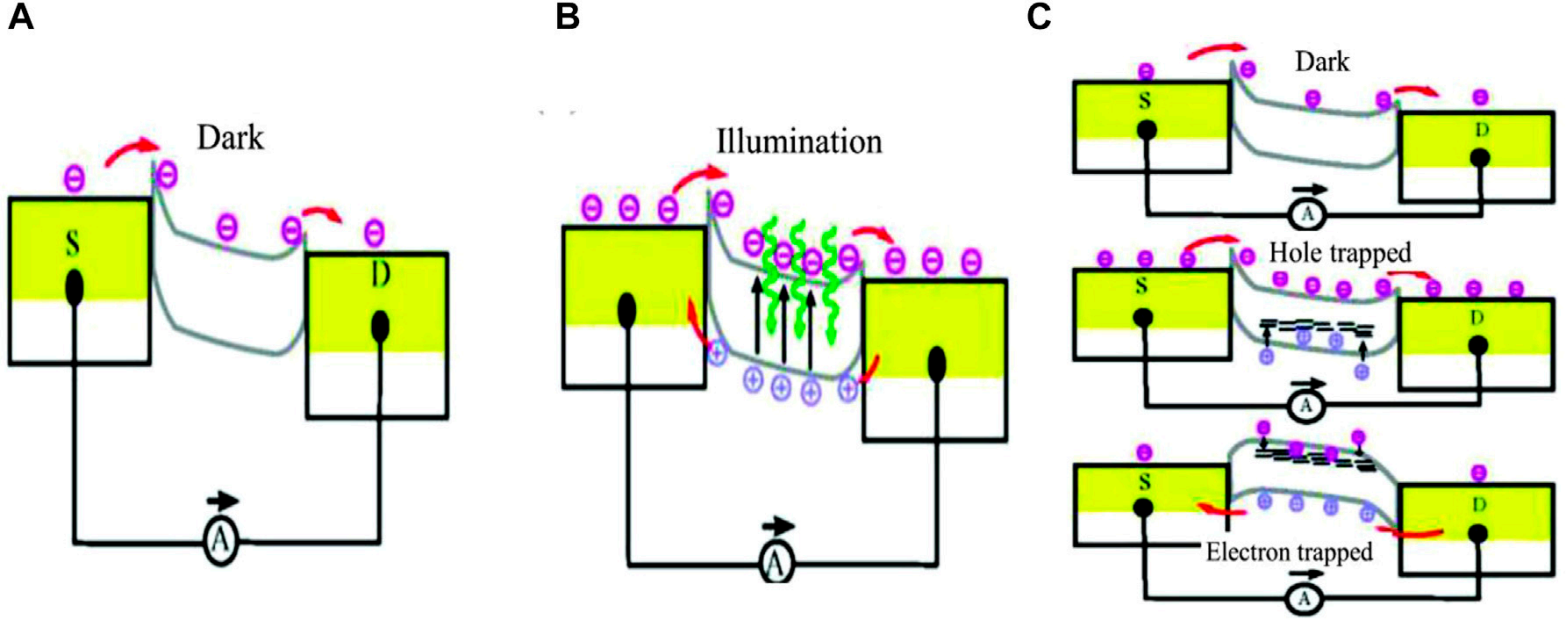
Photodetection mechanisms, **(A**,**B)** Schematic of photoconductive effect (Long et al., 2019), and **(C)** Schematic of photogating effect (Long et al., 2019); Reproduced with permission from (Long et al., 2019).

The photoconductive effect (Long et al., 2019) rises when the conductivity in a semiconductor active material is enhanced by absorption of the specific wavelength of light. Combined charges are separated into electron-hole pairs by incident light in this effect, enhancing the concentration of charge carriers (see Figures 3A,B) (Long et al., 2019). Such induced charge carriers may flow according to the external electric field. This is the mechanism underlying the generation of photocurrent by the photoconductive effect. Recombination of the separated charges may also be possible. Whereas photovoltaic effect-based devices work with zero bias, photoconductor-based devices need an external electric field for their operation in contrast.

#### 2.1.4 Photogating Effect

A photogating effect (Koppens et al., 2014) is the modification of the photoconductive effect, which is usually observed in 2D material-based devices having surface defects. When light is illuminated on active regions having trap sites for surface states, the photogenerated carriers are trapped in those states. Due to such a trapping, it acts as a localized gate and has an impact on carrier density as well as on the conductivity of the active material. This mechanism totally depends on light illumination, where charge carriers are generated through the action of illuminating light, as can be convinced from Figure 3C (Long et al., 2019). The de-trapping of charges is a slow process that enhances the photogenerated carrier lifetime and, hence, the high output current, consequently enhancing the device photo-response performance (Long et al., 2019). This consequence could take place by two mechanisms: one is that electron-hole pairs are generated in the active material by photons and, then, one species of the carriers is trapped there. The alternative process is that one of the two carrier species is formed in the active material molecules or nanoparticles by electron-hole pair generation, which is driven by an internal electric field in the active material. In this situation, the driven charge is re-circulated between the source and drain, while another kind of charge carrier is trapped in the particle or molecule traps. Apart from the surface states or traps, this effect can also be achieved by manipulating the bandgap and regulating the lifetime of different carriers present in the 2D materials (Koppens et al., 2014).

#### 2.1.5 Bolometric Effect

The bolometric effect (Kasirga, 2020) arises from a change of resistivity in an active material as a result of its absorption of illuminated light, as demonstrated in Figure 2C (Qiu and Huang, 2021). The photocurrent generated by heating *via* impinging light increases as a function of applied voltage. The material resistivity in the device can be changed through two processes: 1) variation of carrier mobility attributed to temperature change and 2) change in the amount of carrier current contribution. The important parameters that affect the sensitivity and response time for a device operating on the bolometric effect are the material’s thermal resistance (R_h_) and heat capacity (C_h_) (Kasirga, 2020). In comparison to the photo-thermoelectric effect, the bolometer effect requires external biasing for the generation of photocurrent, while photo-thermoelectric effect-based devices can work without external biasing.

#### 2.1.6 Plasma Wave-Assisted Mechanism

The plasma wave-assisted mechanism for terahertz detection indicates that a field-effect transistor (FET) produces a continuous drain-source voltage when it is biased and exposed by wide spectral radiation (Rupper et al., 2015; Dyakonov and Shur, 1996). Such a voltage has a resonance or non-resonant relationship with the frequency of the illuminated light and is found maximum at plasma oscillation frequency (Rupper et al., 2015). The electron gas in 2D materials acts as a cavity for a plasma wave. When plasma waves are weakly damped in this case, the constructive interference caused by plasma waves can be utilized to enhance the resonance response in the detection (Koppens et al., 2014). However, if the plasma waves are highly damped, detection is available through a wide frequency range, as illustrated in Figure 2D (Qiu and Huang, 2021). Moreover, it was also demonstrated that the potential difference between the drain and source in a FET device contains a DC component, and this DC component allows signal rectification applicable to tetra-hertz radiation detection (Dyakonov and Shur, 1996).

### 2.2 Key Performance Parameters

The responsivity of a photodetector is the ratio of photocurrent to the illuminated light power. Namely, it is described as $R\;=\;I_{p}\;/\;P\;,$ where I_p_ is the photocurrent, which is the difference between illumination current and the dark current (I_L_–I_D_), and P denotes the illuminated light power. In the case of photovoltaic devices, photo-responsivity could also be considered as the ratio of voltage generated by illuminated light (V_p_) and illumination light power (P); $R\;=\;V_{p}\;/\;P$ . The wavelength range between maximum and minimum currents is the spectral response range when responsivity drops by half. The noise equivalent power (NEP) is the minimum power of illuminated light having a signal-to-noise ratio of 1 at the frequency of 1 Hz. This power is a measure related to estimating the detectability of weak optical signals for a photodetector, and can be expressed as; $NEP\;=\;I_{N}\;/\;R$ , where I_N_ denotes the noise current at a frequency bandwidth of 1 Hz.

Quantum efficiency (Q.E.) is the ratio of the number of carriers involved in conducting to the number of incident photons, and can be mathematically defined as; Q.E. =Electron Out/Photon In . The light absorption power at any position z is expressed as $\;P_{z}\;=\;P_{in}\;\left(1\;–\;R\right)\;\left(1-e^{-\alpha z}\right)$ , where P_in_ is the incident light power, R is the material reflectivity, *α* is the material’s absorption coefficient, and position z represents the distance from the surface. The number of photocarriers generated ( η ) by individual photon illumination is of the form $\eta \;=\;\left(1\;–\;R\right)\;\left(1\;–\;e^{-\alpha d}\right)$ *,* where d is the active material thickness having the highest reflectivity R. A photodetector’s detectivity is the reciprocal of NEP; D = 1 / NEP *.* The specific detectivity, on the other hand, is the localized detectivity at a specific unit area with a frequency bandwidth of 1 Hz, which is represented as; $D\;=\;A_{d}B^{1/2}/\;NEP$ , where A_d_ stands for the device area and B is the bandwidth. If the photodetector has greater localized detectivity, it is possible to detect a weak signal efficiently.

The photo-response time of a photodetector is the time required by the device to convert illuminated light into a photoelectric signal. This can also be used as a quantitative measure of the speed of device response. The response time is measured by the rise time (t_r_) and the fall time (t_f_). Rise time is defined as the required time for electrical signals to rise from 10 to 90% of their stable value, whereas the fall time as the time required for electrical signals to fall from 90 to 10% of their stable one. These two times are necessary as a whole in characterizing the capacity of photo-response for a device. The photoconductive gain ( G ) of a device is the ratio of the light-generated charge carrier lifetime and drifts transit time. Accordingly, it reads G = Carrier lifetime / Drift transit time . The quantification of this gain is necessary in order to estimate the ability of photogenerated carriers’ circulation in the channel. The transit time required in the calculation of G depends on carrier mobility, the device channel length, and the applied voltage. Photoconductive gain can also be expressed in terms of light absorbance power P_abs_, such that; $G\;=\;\left(I_{ph}\;/P_{abs}\right)\;\left(h\nu \;/\;q\right)$ , where I_ph_ denotes the photocurrent, P_abs_ is the absorbance power, hν represents the photon energy and q is the unit charge.

## 3 Potential 2D Materials for Photodetection

### 3.1 Graphene

**FIGURE 4:**
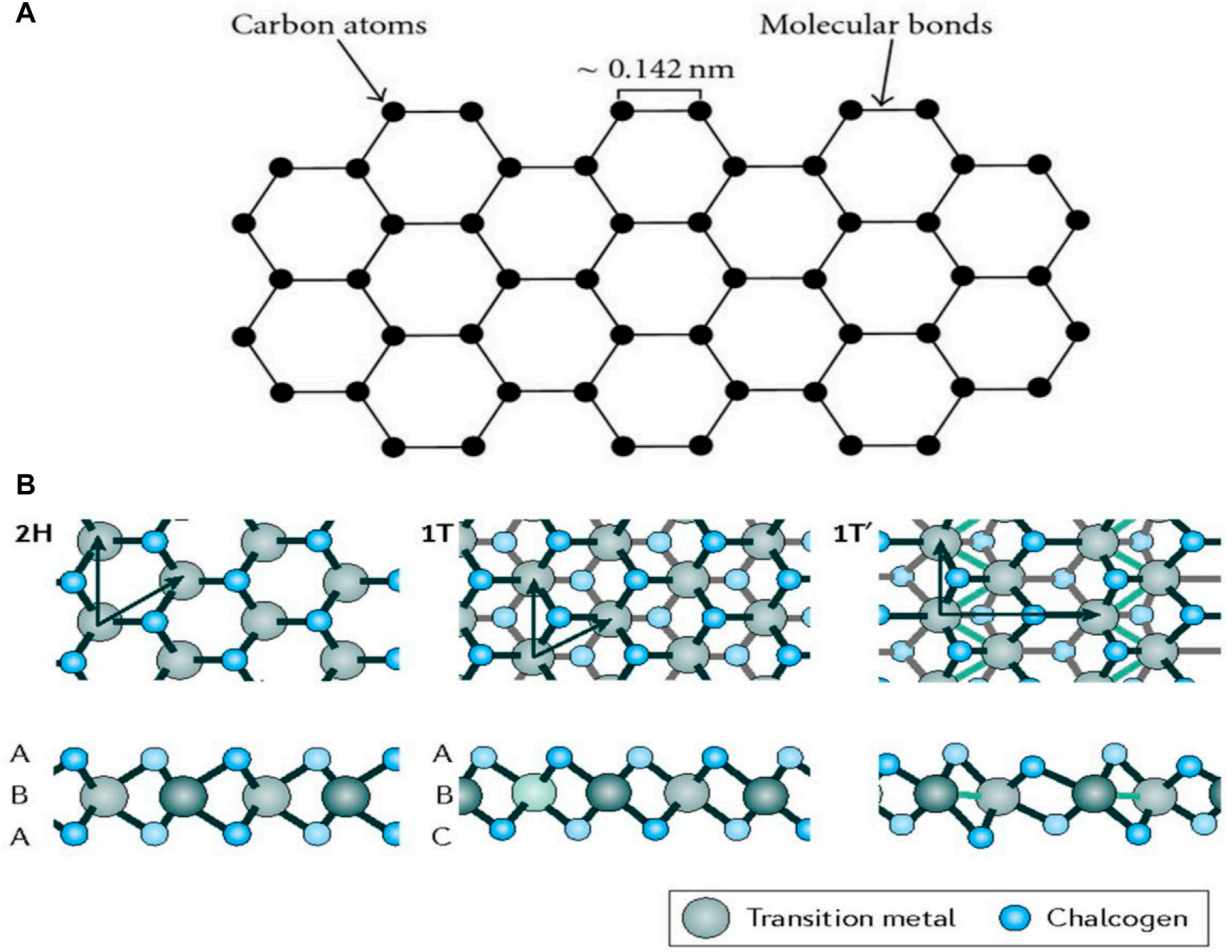
**(A)** Graphene structure (Roberts et al., 2010), and **(B)** Single layered atomic structure of TMDCs in trigonal prismatic (2H) distorted octahedral (1T) and dimerized (1T’) states (Manzeli et al., 2017). Adapted with permission from (Roberts et al., 2010; Manzeli et al., 2017).

Graphene is a two-dimensional allotrope of carbon made up of its sp^2^ hybridization. The hexagonal structure of graphene is depicted in Figure 4A (Roberts et al., 2010). Each atom shown in this figure forms a strong covalent link with three nearby atoms *via* s-bond, where the distance between neighboring carbon atoms is 0.142 nm (Roberts et al., 2010). Graphene’s valance and conduction bands overlap each other at the Fermi level, leading to their strong symmetry (Berger et al., 2004). Thanks to graphene’s single-atom thickness and stable crystalline structure, it exhibits unique qualities, including high carrier mobility (Brida et al., 2013) and a wide response spectrum for light ranging from ultraviolet (UV) to far-IR (FIR), and in terahertz frequencies (Wang et al., 2008). In addition, it is reported that graphene exhibits excellent light-matter interactions (Iqbal et al., 2022), showing an optical absorptivity of 2.3% in the visible and near-IR regions (Nair et al., 2008). Furthermore, selective absorption of specific wavelengths is possible for it by modulating the position of its Fermi level. Consequently, graphene plays an effective role in photodetectors, particularly in the mid-to-far-infrared range.

### 3.2 2D Transition Metal Dichalcogenides

TMDCs are semiconductor materials that have layered structures consisting of single or multilayer with hexagonal symmetry. The generic chemical formula of TMDCs is MX_2_ where M = Mo, Re, and W, whereas X = S, Te, and Se. Concerning this, distinct TMDCs have identical crystal structures, such as MoS_2_, WS_2_, and MoSe_2_. Figure 4B (Manzeli et al., 2017) represents the three major structural phases of TMDCs: trigonal prismatic (2H), distorted octahedral (1T), and dimerized (1T’). Many of them, in particular, are monolayer honeycomb-like patterns that are further subdivided into other categories. Trigonal prismatic structures with D6h group symmetry and distorted octahedral structures with D3h group symmetry are named honeycomb (2H) and centered honeycomb (1T) structures, respectively (Ataca et al., 2012; Svetin et al., 2014; Manzeli et al., 2017). Through diverse mixtures of chalcogenide elements with transition metals, there are roughly 40 different TMDC material types. They offer a range of their novel features, including specific charge density wave (Svetin et al., 2014), semimetal nature (Shishidou et al., 2001), and efficient superconductivity (Xi et al., 2016). When relevant layers expand, for instance, the direct bandgap of about 1.8 eV for monolayer MoS_2_ can transform into a bulk semiconductor with a 1.2 eV indirect bandgap (Splendiani et al., 2010). For the case of group V layered dichalcogenides like TaS_2_, TaSe_2_, and NbSe_2_, there exist charge density waves (CDW) with a phase within the materials. Such waves are caused by layers in the material, biasing, deformation of substrates, and/or current stimulation. Some other topological semimetals, such as PtSe_2_, PdTe_2_, and PtTe_2_, have exceptional chiral coupling transportation capabilities together with nonlinear optical features (Fei et al., 2017). They also exhibit other odd physical properties, such as distinctive exceptional carrier characteristics and the ability of wideband photo-detection. Such properties are mainly owing to their symmetrically shielded electronic state. Apart from other considerable features, there is broad-range of scientific branches where TMDCs are applicable. Some of them are optoelectronics, configurable excitonic devices, and spin-valley lasers (Ye et al., 2016a).

### 3.3 Black Phosphorous

**FIGURE 5:**
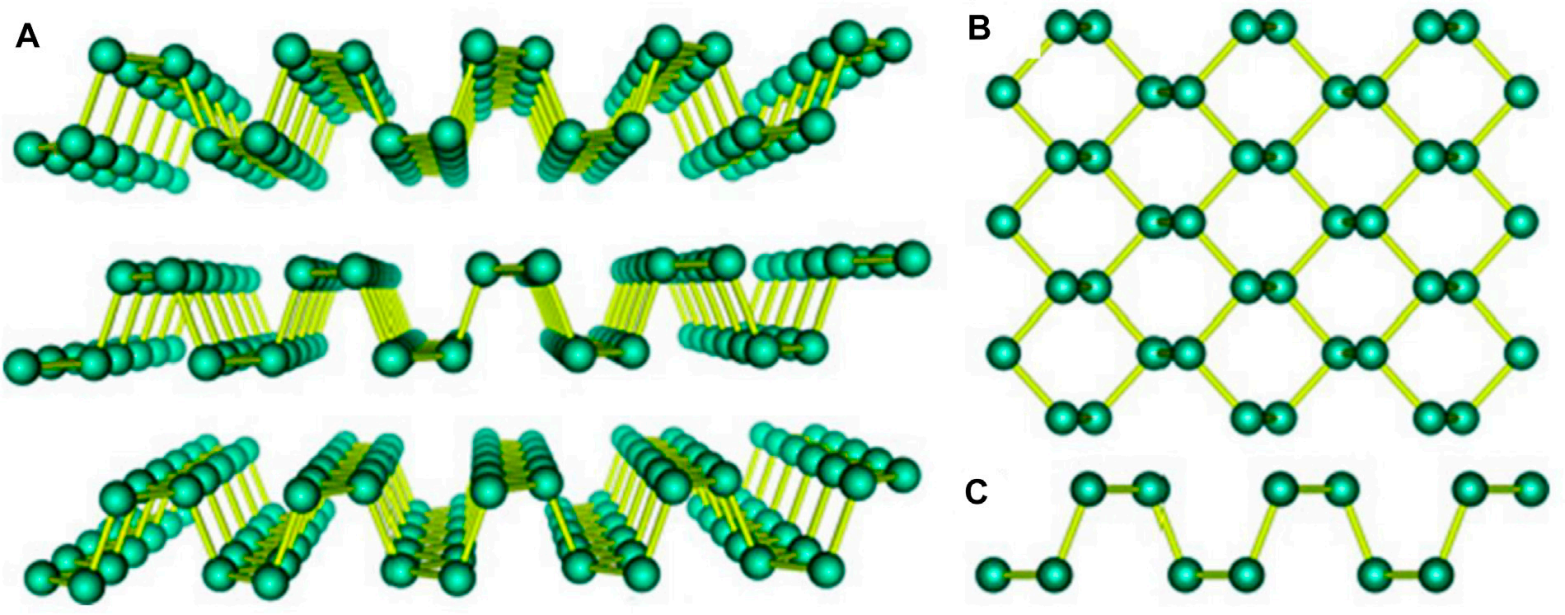
**(A)** Multilayered crystal structure of black phosphorus (Shao et al., 2014), **(B)** Top view (Shao et al., 2014), and **(C)** Side view (Shao et al., 2014); Adapted with permission from (Shao et al., 2014).

The currently discovered black phosphorus (BP), having a thickness-dependent bandgap of 0.3–1.5 eV, is a new member of the layered materials family. This material can be potentially used as an ingredient of efficient optoelectronic devices in a wide IR spectral region. BP 2D sheets are appropriate for monolithic integration with standard electronic materials like silicon, and may be deposited on a variety of substrates, including flexible ones (Youngblood et al., 2015). BP nanosheets have a much high optical absorption than single-layered other 2D materials, and it increases with a decrease in number of layers (Zhang et al., 2020; Qiao et al., 2014). On one hand, to detect faint optical signals using the conventional BP photodetectors and solar cells is a challenge because the responsivity of such devices is low typically. Previous research associated with this has addressed only the visible and near-infrared wavelength ranges as far as we know (Liu et al., 2018; Ye et al., 2016b). Black phosphorus crystals, which are puckered layers of horizontally piled phosphorus atoms, retain less crystalline symmetry than graphene. Due to this, their band structure is quite asymmetric and this appearance is in fact linked with anisotropic electronic and optical conductivity. Multilayered 2D structure of black phosphorus is displayed in Figure 5 (Shao et al., 2014). The aforementioned asymmetry in the crystalline structure also affects properties of carrier transport in materials (Wang and Lan, 2016). Recent research on BP-based photodetectors has mainly devoted to the improvement of responsivity, increment of spectral detection range, suppressing of dark current, measuring of time response, enhancement of polarization sensitivity, and realization of waveguide integration.

### 3.4 Group V-Based 2D Materials

It is expected that next-generation industry of group V element-based 2D materials would grow highly with the prospects of their wide electronic application. This outlook is owing to the extraordinarily high carrier mobility and adjustable bandgap of those materials. The flexibility of their bandgap is indeed in contrast to graphene’s zero bandgap and TMDCs’ indirect bandgap that is implicated with low mobility of carriers (Britnell et al., 2013; Zhang et al., 2016a). The crystal lattice of BP, shown in Figure 5 (Shao et al., 2014), has a direct bandgap that can effectively be paired with light. This preferable property of BP lends itself to optoelectronic applications with a viable choice, such as photodetectors, sensors in the visible to MIR range, and laser technologies, in addition to its wide range nanophotonic applications (Iqbal et al., 2021c). 2D tricyclic arsenene also exhibits excellent features in this context, such as high stability (Ma et al., 2016) and tenability of bandgap (Zhang et al., 2016a) that are, apparently, advantageous to its optoelectronic-device applications. The efficient electrical transport capabilities of 2D arsenic material can also be influenced by the variation of external electric fields. Owing to its puckered monolayer frame analogous to black phosphorus, 2D arsenic material displays a non-negligible anisotropy and this peculiarity is in effect more noticeable than that of BP. It is also known that the arsenic material is more resilient than 2D multilayered phosphorus. This acts as a merit in preparing and applying such ultra-thin layer samples over huge areas. The electrical transport properties of antimony in bulk are similar to those of metals, wherein the indirect bandgap of 2D layered antimony (Zhang et al., 2016a) may impede the evolution of optoelectronics. Its strong carrier density and mobility, however, make it a notable research weightage. According to existing research, arsenic and antimony have attracted a lot of attention as new 2D stacked semiconductor materials in device applications such as transistors, quantum spin devices, optoelectronic devices, and many more (Zhang et al., 2016a; Xie et al., 2016).

### 3.5 Group III-V Based 2D Materials

GaSb, InSb, AlSb, InAs and their trio, quadri, along with quaternary alloys, are among the III–V semiconductors known as antimonides. These materials have a wide range of band alignments, ranging from type I to type III (Vurgaftman et al., 2001). Whereas electrons and holes, for the case of type I, are restricted in the same material, one material’s conduction band is positioned below the valence band of the next for the case of type III. The III-Sb multilayered materials have a wide bandgap, spanning from 0.1 to 2 eV, and are approximately lattice-matched with GaSb. The heterostructure materials of quaternary alloys, for instance, AlGaAsSb and GaInAsSb, have wide and narrow bandgap, and constitute the foundation of MIR III-Sb LEDs. The aforementioned characteristics, which distinguish III–Sb compounds from other III–V semiconductors, provide unequalled chances for bandgap and small band offset engineering. This enables us to create artificial man-made materials with an effective bandgap that may be tuned through an appropriate design throughout the MIR range. It is expected that such a manufacturing is being a promising technology for developing inter-band MIR response devices on the basis of using type I or type II heterostructures.

Furthermore, germanium, which is easily accessible in complementary metal-oxide-semiconductor (CMOS) foundries, can be used to improve hole-mobility in compressively stressed p-channel transistors. This is the reason why the present integrated photodetectors are frequently built on it. Owing to the specific nature of Ge, photodetectors based on the scalable Ge can be efficiently used with a level of maturity through a sensing frequency range surpassing 67 GHz and operating at 100 GBd (Gigabauds) (Chen et al., 2016). However, due to the indirect nature of Ge bandgap, they frequently result in excessive dark currents and weak capacity of absorption. By the way, most of group III–V materials have larger absorptivity and their direct adjustable bandgap allows them to be operated suitably as efficient optical sensing devices, like lasers (Staudinger, 2020) or LEDs (Li et al., 2019).

### 3.6 2D Halide Perovskite Materials

MAPbI_3_, CH_3_NH_3_PbI_3_, CsPbBr_3_, and FAPbI_3_ are halide perovskite materials with the chemical formula ABX_3_ that have attracted a lot of interest because of their exceptional photoelectrical performance, which includes extremely efficient photoluminescence and absorbance (Guan et al., 2021). The cubic perovskite structure is transformed into a 2D layered structure by replacing the A site cation with a long-chain organic group, in which the 2D halide perovskites are held together by ionic bonds in nature, and their bandgap may be tuned by varying chemical compositions, which is different from conventional 2D materials. 2D halide perovskites have stronger moisture resilience than cubic halide perovskites due to the hydrophobic feature of the long-chain surface group, which, together with their superior optical properties, makes them desirable for high-gain photodetector applications.

## 4 Synthesis of 2D Materials: An Overview

**FIGURE 6:**
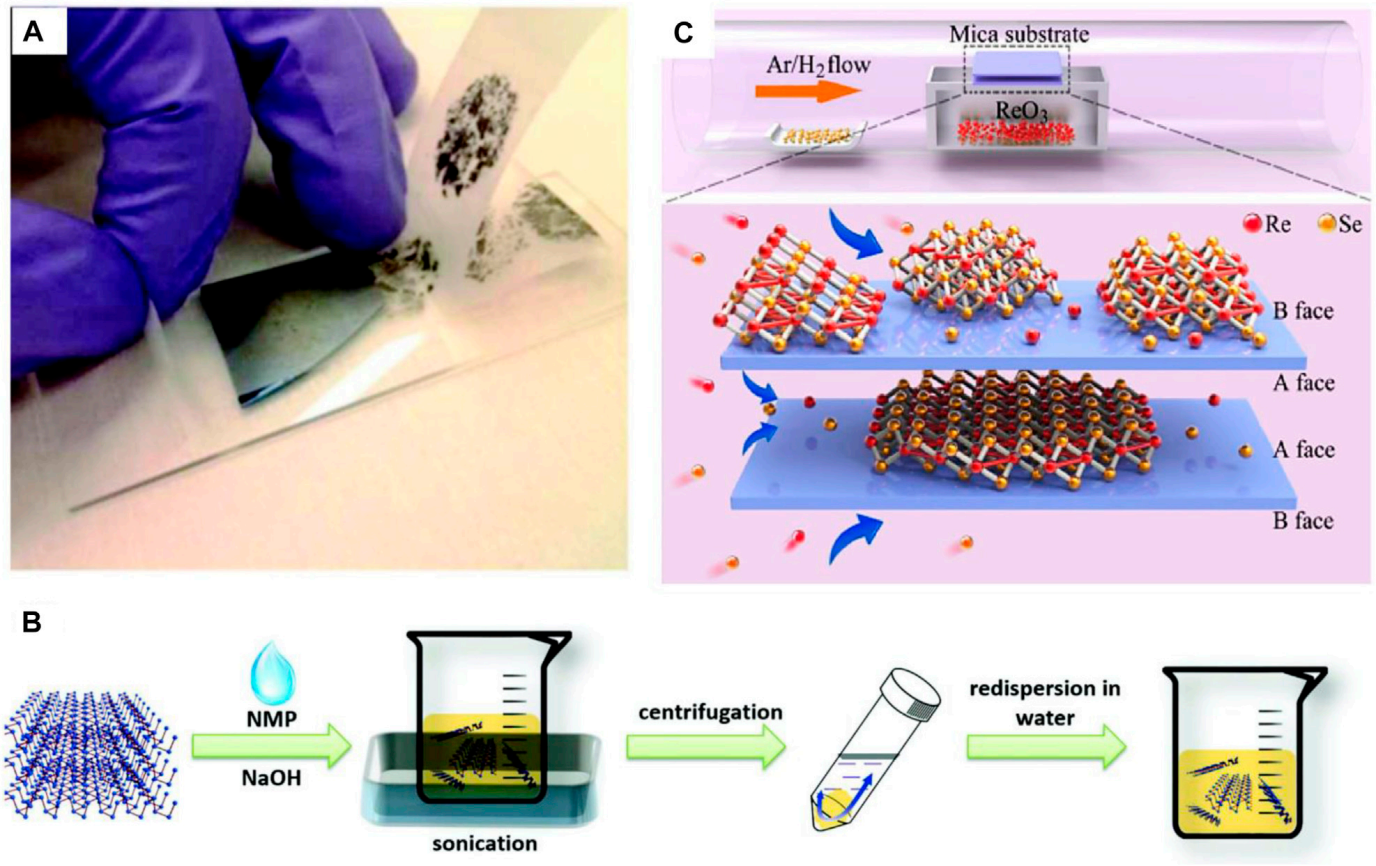
Schematic of 2D material synthesis methodologies **(A)** Mechanical exfoliation technique (Huang et al., 2015), **(B)** Liquid phase exfoliation (Guo et al., 2015), and **(C)** Chemical vapor deposition (Cui et al., 2017); Reproduced with permission from (Guo et al., 2015; Huang et al., 2015; Cui et al., 2017).

The technique for fabricating high-quality 2D materials is critical to the device’s performance. Many originative methodologies for synthesizing 2D materials, including micromechanical exfoliation, liquid-phase exfoliation, epitaxial growth, and chemical vapor deposition (CVD), have been developed so far. Micromechanical exfoliation is a physical method for obtaining optimized 2D materials by, especially, synthesizing single-layered graphene (Novoselov et al., 2004), multi-layered MoS_2_ (Miremadi and Morrison, 1987) and WS_2_ (M and Late, 2014). Though this method can be conveniently used along this line that regards small samples, it is still limited from the viewpoint of an industrial level. A schematic of mechanical exfoliation method is shown in Figure 6A (Huang et al., 2015).

On the other hand, the liquid-phase exfoliation technique can be applied to syntheses at an industrial level, because that technique is suitable for producing a large number of samples. To ensure high quality for the 2D materials in this method, materials should be dispersed in specified solvents and the materials’ layers need to be peeled out on demand by using ultrasonic energy as depicted in Figure 6B (Guo et al., 2015). Coleman et al. were the first to report a 2D material produced by liquid exfoliation using an organic solution that has a high boiling point. Solvent is not removed from the required 2D layer in liquid exfoliation method, but it is compromised with device performance (Alzakia and Tan, 2021). Pan et al. synthesized WS_2_ thin film using such liquid exfoliation method (Pan et al., 2016); a shortcoming of their peeling procedure is that it is somewhat less efficient due to the material instability in water. It is reported that, in addition to exfoliation techniques, CVD can be used for preparing a uniform and high-quality sample in a large area [see Figure 6C (Cui et al., 2017)]. Cui et al. reported an effective way about synthesizing ReSe_2_ thin film based on the CVD method with the controlling of parameters (Cui et al., 2017). CVD method is advantageous in thin film syntheses over other techniques of syntheses owing to its simplicity in operation, high repeatability, and high-quality. As a result, this method is very useful in achieving a variety of specific device based applications.

## 5 Overview of 2D Materials-Based Photodetectors

### 5.1 Graphene Based Photodetectors

Graphene is a 2D material having hexagonal geometry and exhibiting optical absorption in a narrow region in the light-impinging direction because its thickness is ultra-thin. Due to this, its photoresponsivity is limited to some extent, where it is given by 0.5 mAW^−1^ with a bandwidth of 500 GHz (Mueller et al., 2010). However, it is reported that metal-graphene-metal photodetectors with asymmetric electrodes exhibit a high responsivity of 6.1 mAW^−1^. This consequence is in contrast to graphene’s high speed, circuit compatibility, and applicability in a wide frequency range (Lou et al., 2016). Various strategies for improving optical absorption for graphene photodetectors have been proposed up to now. One of them is to use nanostructured plasmonics. While this procedure increases light concentration, it is adaptable for accomplishing multicolor detection together with the boosting of the device’s quantum efficiency. It has also been reported that the quantum efficiency of a graphene photodetector with plasmonic nano-antennas sandwiched within the graphene layers is up to 20%, whereas the device’s operating bandwidth can be shortened by regulating the nanostructures’ resonance (Fang et al., 2012). As another strategy to enhance photoresponsivity, there is a method of integrating quantum dots (QDs) into graphene. Based on this method, photoresponsivity in photodetection is higher and reported to be 10^7^–10^8^ AW^−1^ (Konstantatos et al., 2012). This facilitates the passage of photo-excited carriers to the graphene layer, whilst the opposite carriers are trapped according to the field-effect doping phenomenon. PbS QDs have also been used in graphene photodetectors, where their photoresponsivity is reported as 10^7^ AW^−1^ (Sun et al., 2012). Another effective approach is the use of graphene-QD-based photodetectors that have low operational speed and a limited bandwidth in micro-cavities for optimized photoactivity in graphene (Gan et al., 2013). The response speed of this device is relatively fast. Additionally, it also exhibits high efficiency, ultra-broad bandwidth, and enhanced light responsivity. However, the device size is larger than conventional photodetectors, as its major drawback (Kim et al., 2011).

**FIGURE 7:**
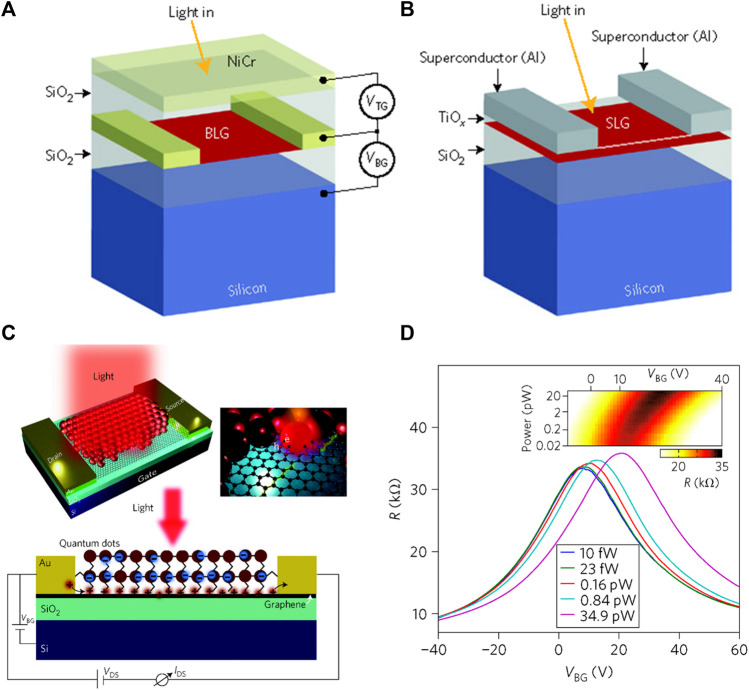
**(A)** Graphene bi-layer bolometric device structure (Koppens et al., 2014), **(B)** Graphene–Al tunnel junction bolometer device structure (Koppens et al., 2014), **(C)** Photodetectors based on QDs (Konstantatos et al., 2012), and **(D)** Resistance–voltage relationship for the graphene–QD structure (Konstantatos et al., 2012); Reproduced with permission from (Konstantatos et al., 2012; Koppens et al., 2014).

By biasing single-layer graphene (SLG) at ambient temperature, the associated bolometric reactions were examined. This reveals two processes that lead to change polarized bolometric photocurrents: (1) light-induced carriers increase conductance, and (2) temperature dependence of carriers reduces conductance. In these mechanisms that are regulated by changing electric field, photovoltaic (PV) effects dominate where the carrier density is significantly low. For the case of room-temperature graphene bolometric detectors, photo-thermoelectric effects (PTE) dominate further, whereas a short photoresponsivity of 0.2 mAW^–1^ was observed (Freitag et al., 2013). To open a bandgap and provide Te-dependent resistance in order to address this issue, Yan et al. (Yan et al., 2012) employed a bi-layer graphene (BLG) which is comprised of a dual gate design with an anti-reflective top gate (Figure 7A) (Koppens et al., 2014). The device was measured with a four-terminal arrangement under MIR exposure (10.6 m), whereas the light response was identified through a bolometric mechanism (Liu et al., 2013). The detector showed an innate spectrum of more than 1 GHz and a NEP of 33 fWHz^−1/2^ at a temperature of 5 K, which is much smaller than that of conventional bolometers. Another method for achieving thermal resistance in graphene is to integrate the electrical system within the graphene by adding disorders. This can be obtained from a graphene–superconductor tunnel junction bolometer composed with defective graphene films as shown in Figure 7B (Koppens et al., 2014). Usually, the analysis associated with this system is carried out with radio-frequency waves, but is also valid for optical detection (Vora et al., 2012). Graphene is a good option for room-temperature photodetection purpose because of its several virtues which are greater mobility, less carrier transit time, and good susceptibility to the electrostatic disturbance caused by photogenerated surface carriers. Colloidal QDs synthesized by PbS (Sun et al., 2012; Jeong et al., 2020) shown in Figure 7C (Konstantatos et al., 2012) are an example of graphene-sensitized light-absorbing particles. Photon detection at weak incident light intensities, especially at single-photon levels, necessitates a gain mechanism capable of providing numerous electrical carriers for each individual illuminated photon. This can be accomplished by tuning surface specifically in a way that it absorbs light efficiently prior to the transmission of charge carriers into the conductor. This is accompanied by a shift of resistance versus gate-voltage curve as shown in Figure 7D (Konstantatos et al., 2012).

### 5.2 Transition Metal Dichalcogenides Based Photodetectors

**FIGURE 8:**
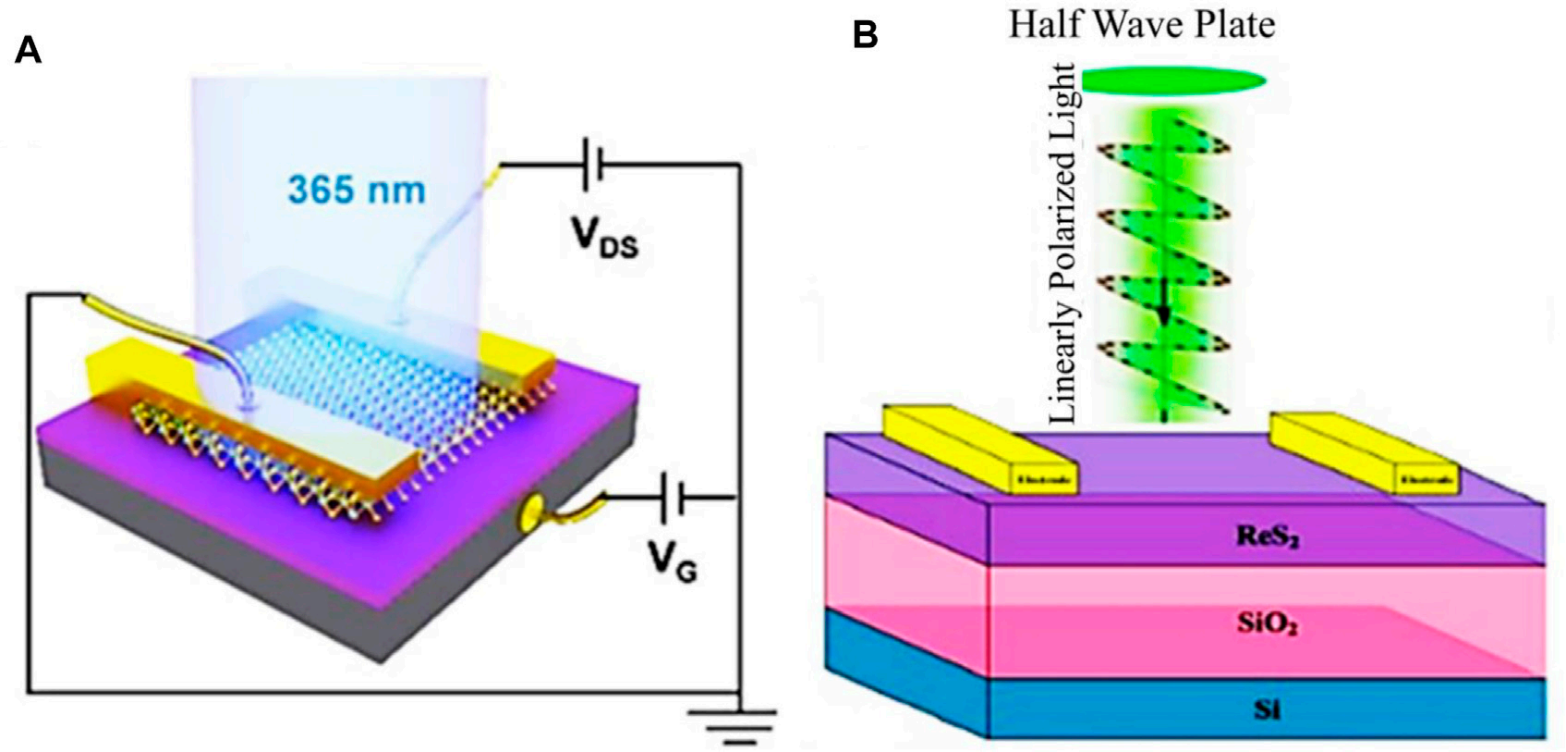
**(A)** Schematic of MoS_2_ single-layered phototransistor (George et al., 2021), and **(B)** Schematic of ReS_2_ photodetector (Liu et al., 2016a); Reproduced with permission from (Liu et al., 2016a; George et al., 2021).

TMDCs, such as MoS_2_, MoSe_2_, WS_2_, WSe_2_, RS_2_, RSe_2_, and MoTe_2_, are potential candidates for materials applicable to photodetection. The MoS_2_ monolayered and few-layered structures have attractive electronic properties available for optoelectronic applications, such as high charge carrier mobility of about 200 cm^2^V^−1^s^−1^, direct bandgap of 1.8 eV, efficient current on/off ratio, chemical and mechanical stability, as well as a stronger light-matter interaction (Splendiani et al., 2010; George et al., 2021; Ponomarev et al., 2015). George et al. reported a long-living giant persistent photoconductor (GPPC) in FET from CVD fabrication of monolayer MoS_2_ at an operating wavelength of 365 nm. This improvement was thanks to the conductivity enhanced up to the factor of 10^7^ (schematic for this device is shown in Figure 8A (George et al., 2021)). A conventional transistor configuration of MoS_2_ was also reported by Yin et al. where they used MoS_2_ nanosheet as an effective region. They showed that this transistor exhibits good efficiency at an operating wavelength of 670 nm with a bandgap of 1.8 eV in addition to the responsivity of 75 mAW^−1^ with a 50 ms response speed (Yin et al., 2012). Lopez-Sanchez et al. reported an enhanced configuration of the same MoS_2_ single-layered device, where they observed an increased responsivity of 800 mAW^−1^ at 680 nm of operating wavelength. This high responsivity is owing to the enhanced carrier mobility in their analysis and to their improvement in the positioning methodologies (Lopez-Sanchez et al., 2013). They also reported a low NEP of the order of 1.5 × 10^−15^ WHz^−1/2^, where it is linked to the minimized dark current which, in turn, associated with MoS_2_ low bandgap. While this minimizes the thermally excited carriers, it was reported that the response time of the device is slow. However, the undesirably longer response time can be reduced by removing the trap charges with the help of short pulses on gate terminals. The sublinear photocurrent of this device is largely dependent on the intensity of incident light. This indicates that the trapping of charge carriers at MoS_2_ or at the MoS_2_-SiO_2_ interface plays an essential part in device sensing activity.

MoSe_2_ also possesses attractive features like MoS_2_, such as effective photoluminescence, efficient exciton binding energy, as well as a direct bandgap of 1.5 eV (Zhang et al., 2014a). The advance of synthesis techniques for this material, such as chemical vapor deposition (CVD) and exfoliation techniques, have improved device activity and photodetection applications (Abderrahmane et al., 2014; Lu et al., 2014) Wang et al. summarized 2D materials for photodetection applications (Wang et al., 2018), while Xia et al. investigated MoSe_2_-based photodetector (Xia et al., 2014a). Zhang et al. synthesized MoS_2_ monolayer based phototransistor using a CVD method, and their fabricated devices exhibit responsivity more than MoSe_2_ atomic layers with a few orders of mAW^-1^ (Zhang et al., 2013a). On the other hand, multilayered MoSe_2_ devices grown by the CVD method show an improved responsivity but with a degraded response speed (Jung et al., 2015). Abderrehmane et al. reported a few-layered MoSe_2_ photodetector framed using the mechanical exfoliation method (Abderrahmane et al., 2014); they demonstrated that it exhibits an enhanced responsivity of 97.1 AW^-1^ at 532 nm wavelength and a response time of tens of milliseconds. Another type of TMDC material, so-called the WS_2_ photodetector, has also been synthesized using several techniques (Sik Hwang et al., 2012). Perea-López et al. (Perea ‐ López et al., 2013) studied the few-layered WS_2_ photodetectors synthesized by CVD method. They showed that their detectors exhibit a dependency on photon energy, where the measured device responsivity and response speed were 92 µAW^−1^ and 5 ms, respectively at an operating wavelength range of 457–647 nm. In addition, WSe_2_ with its single and multi-layered structure has been investigated for photodetection applications. Zheng et al. synthesized monolayered WSe_2_ device by CVD technique and explored the effect of the variation of the work function in metal contact on device performance; they exhibited a maximum responsivity of 1.8 × 10^5^ AW^−1^ with the Pd contact and minimum device responsivity with the Ti-contact at the operating wavelength of 650 nm (Zhang et al., 2014b). The least-responsive metal contact (Ti) showed an efficient response time of 23 ms. This response time is larger than its counterpart and attributed to the Schottky contact between active materials. Hence, along this line, it may be necessary to improve metal contacts regarding on device performance.

An advanced 2D material MoTe_2_ has gained intense attention owing to its interesting optoelectronic features (Lin et al., 2014). However, the Re-based dichalcogenides are different from the conventional ones because of their high crystallinity. ReS_2_ and ReSe_2_ are anisotropic semiconductors with efficient electrical, optical, and mechanical anisotropies, as well as a layer-dependent bandgap and carrier mobilities that contribute to altered device performance (Wilson and Yoffe, 1969; Yang et al., 2014). A recent report showed that ReSe_2_ device has a response of 95 AW^−1^ along with a short response time at an operating wavelength of 633 nm (Yang et al., 2014). As discussed earlier, photo-detective devices are environmentally sensitive in which the charge transfer between active material (ReSe_2_) and the environment directly influences the device performance by altering the doping and hence altering the carrier lifespan (Jo et al., 2016). Encapsulation passivation can be used to limit the device dependence on its surroundings in order to enhance its efficiency. Although ReSe_2_ is an effective device for photodetection, it has a drawback which is that, in the dark situation, the flowing current does not return to the primitive dark current level. This aspect is attributed to the slow recombination of charge carriers, which can be confirmed by resetting the device *via* exerting short pulses at the gate terminals (Konstantatos et al., 2012; Ahn et al., 2021). For the case of detecting polarized light with ReS_2_ photodetectors, the reported responsivity is somewhat enhanced and ranged from 3.97 × 103 to 1.18 × 106 AW^-1^ at an operation wavelength of 1064 nm. This enhancement is caused by electron doping as demonstrated in Figure 8B (Liu et al., 2016a).

**FIGURE 9:**
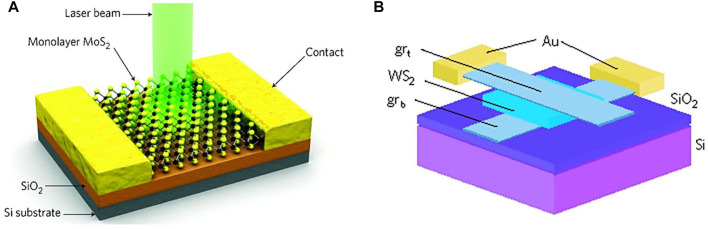
**(A)** Schematic of MoS_2_ based photodetectors (Lopez-Sanchez et al., 2013), and **(B)** Schematic of WS_2_ based photodetectors (Koppens et al., 2014); Reproduced with permission from (Lopez-Sanchez et al., 2013; Koppens et al., 2014).

In comparison with existing conventional direct-bandgap semiconductors, TMDCs can also play an additional role in flexibility and in the simplification of device processing. TMDC devices, which act as photodiodes or photoconductors, usually operate under proper biasing. For instance, Lopez-Sanchez et al. showed that the monolayered MOS_2_ based device exhibits a responsivity of 880 AW^−1^ and a high response time of 9 s as depicted in Figure 9A (Lopez-Sanchez et al., 2013). On the one hand, vertical transport between graphene electrodes separated by various TMDCs has been reported, where it was demonstrated that the graphene acts as a tunable electrode in this case whereas the TMDCs act as photoactive materials. Herein, an efficient current was produced through the creation of electron-hole pairs at TMDCs under the separation at graphene electrode layers, exhibiting a responsivity of 0.1 AW^−1^ and a quantum efficiency of 30% as illustrated in Figure 9B (Koppens et al., 2014). The WS_2_ based flexible photodetectors were reported at the operating wavelength of the visible to IR ranges with a high photo-responsivity of 4.04 mAW^−1^ (Li et al., 2020). Other WS_2_-graphene combinations, such as Graphene-WS_2_-Graphene, have also been reported at an illumination wavelength of 0.532 µm (Pham et al., 2022). This report showed an increased detectivity and photoresponsivity of 10^11^ jones and 3.5 AW^−1^ respectively for the Graphene-WS_2_-Graphene combination.

It is known that the photodetection performance of the TMDCs is excellent, with the general estimation of their responsivity range as 10^−7^–10^7^ AW^−1^ whereas the range of their response time is estimated as 10^−5^–10^3^ s from literature studies (Yin et al., 2012; Lopez-Sanchez et al., 2013; Perea ‐ López et al., 2013; Abderrahmane et al., 2014; Zhang et al., 2014b; Pham et al., 2022). In addition, along this line, an enhancement of the responsivity was also observed at the material trap site or at the material-dielectric interface. The response speed has an inverse relationship with trap sites usually. Along with the aforementioned TMDCs-based photodetectors, Pham et al. described various other single and multi-layered photodetectors with their specific operating conditions and parameters (Pham et al., 2022). Moreover, synthesis methods, the surrounding environment, and the number of layers is also important factors that should be considered in enhancing the device’s performance.

### 5.3 Black Phosphorus Based Photodetectors

**FIGURE 10:**
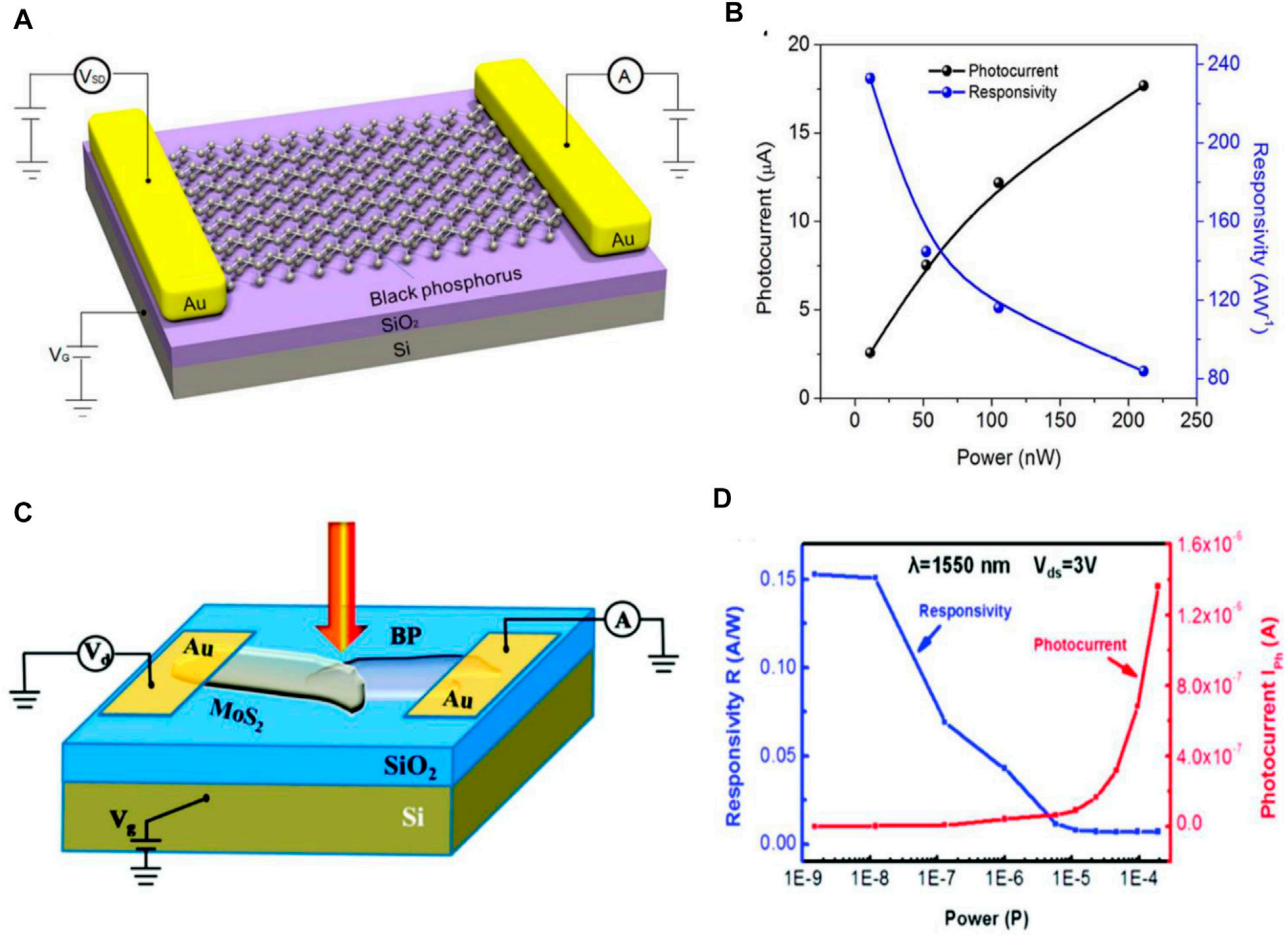
**(A)** Schematic of black phosphorus-based photodetector (Yan Liu et al., 2018), **(B)** BP photodetector responsivity (Yan Liu et al., 2018), **(C)** Schematic of BP-MoS_2_ photodetector (Ye et al., 2016b), and **(D)** BP-MoS_2_ photodetector responsivity (Ye et al., 2016b); Reproduced with permission from (Ye et al., 2016b; Yan Liu et al., 2018).

Black phosphorus (BP) is a thermodynamically stable form of phosphorus with −395 KJmol^-1^ of formation energy at room temperature. The exterior, structure, and properties of this material are similar to those of graphite which has a puckered structure. However, unlike graphene, BP is a kind of semiconductor that has a direct band and reveals anisotropic light-matter interactions. This proves that BP is a good candidate for optoelectronic applications. It is observed that this material exhibits an easier transit of carriers, whereas their scattering phonons are minimized. Liu et al. investigated the black phosphorus photodetector at the operating range of the wavelength from visible to near IR (1550 nm). Through their analysis, an enhanced responsivity of up to 230 AW^−1^ with decreasing wavelength is demonstrated [see Figures 10A,B (Liu et al., 2018)]. Additionally, Lei et al. analyzed a BP-MoS_2_ based photodetector and they showed that it exhibits ultrahigh photo-responsivity of 153 AW^−1^ at the wavelength of 1.55 µm with a short response time of 15 µs (Figures 10C,D) (Ye et al., 2016b).

Chen et al. (Chen et al., 2017) reported a BP-based photodetector in which the BP is sandwiched between hBN layers, including its detection characteristics in the infrared region. They discovered that, when the wavelength and vertical electrical biasing were increased, device’s light absorption was reduced due to a decrease in the bandgap. This appearance is responsible for the degradation of device performance and can be attributed to higher concentrations of carriers and lower carrier lifetimes. An advantage of the hBN layer is that it provides a favorable interface with the reduction of black phosphorus oxidation. Keng et al. (Kang et al., 2017) studied photodetectors made by black phosphorus doped with n-type and p-type impurity components, where the dopant concentrations depend on the BP layer thickness. They demonstrated that such devices on a transparent substrate show the responsivity of 1.4 × 10^4^ AW^−1^ for a BP thickness of 10 nm (Kang et al., 2017). However, the enhanced scattering of carriers with phonons by IR light illuminance affects the carrier mobility and thereby also the device performance. These consequences should be considered for realizing efficient black phosphorus-based photodetectors.

### 5.4 2D Halide Perovskites Based Photodetectors

Photodetection in the visible region is improved by most halide perovskites having a significant bandgap of >1.3 eV. Photodetection with 2D layered perovskites such as (C_4_H_9_NH_3_)_2_PbBr_4_ crystals has shown a high responsivity of around 2100 AW^−1^ (Guan et al., 2021). Furthermore, a very high detectivity of 10^13^ Jones was reported using 2D (C_6_H_5_C_2_H_4_NH_3_)_2_PbI_4_ perovskite with ligand-engineering and channel length reduction into the nano-scales (Guan et al., 2021). Xu et al. used a low-temperature solution-based approach to synthesize MA_0.5_FA_0.5_Pb_1−x_Sn_x_I_3_ perovskite in 2017, and they discovered that by increasing the amount of Sn in the perovskite, the bandgap can be gradually lowered to 1.17 eV, which is suitable for NIR photodetection (Xu et al., 2017). Sn doping in 2D perovskite has been used for the first time to increase the detection range from the visible to the NIR regime. Wang et al. successfully fabricated an IR photodetector based on (n-C_4_H_9_NH_3_)_2_PbI_4_/(n-C_4_H_9_NH_3_)_2_(CH_3_NH_3_)Pb_2_I_7_ 2D perovskite heterostructure in 2019, which realized direct detection at 980 nm with a responsivity of 10^7^ AW^−1^ (Guan et al., 2021). In general, given the strong tunability of the material bandgap and the advancement of device design, it appears that more 2D perovskite devices for IR applications will be produced in the near future.

### 5.5 2D Heterostructure Based Photodetectors

**FIGURE 11:**
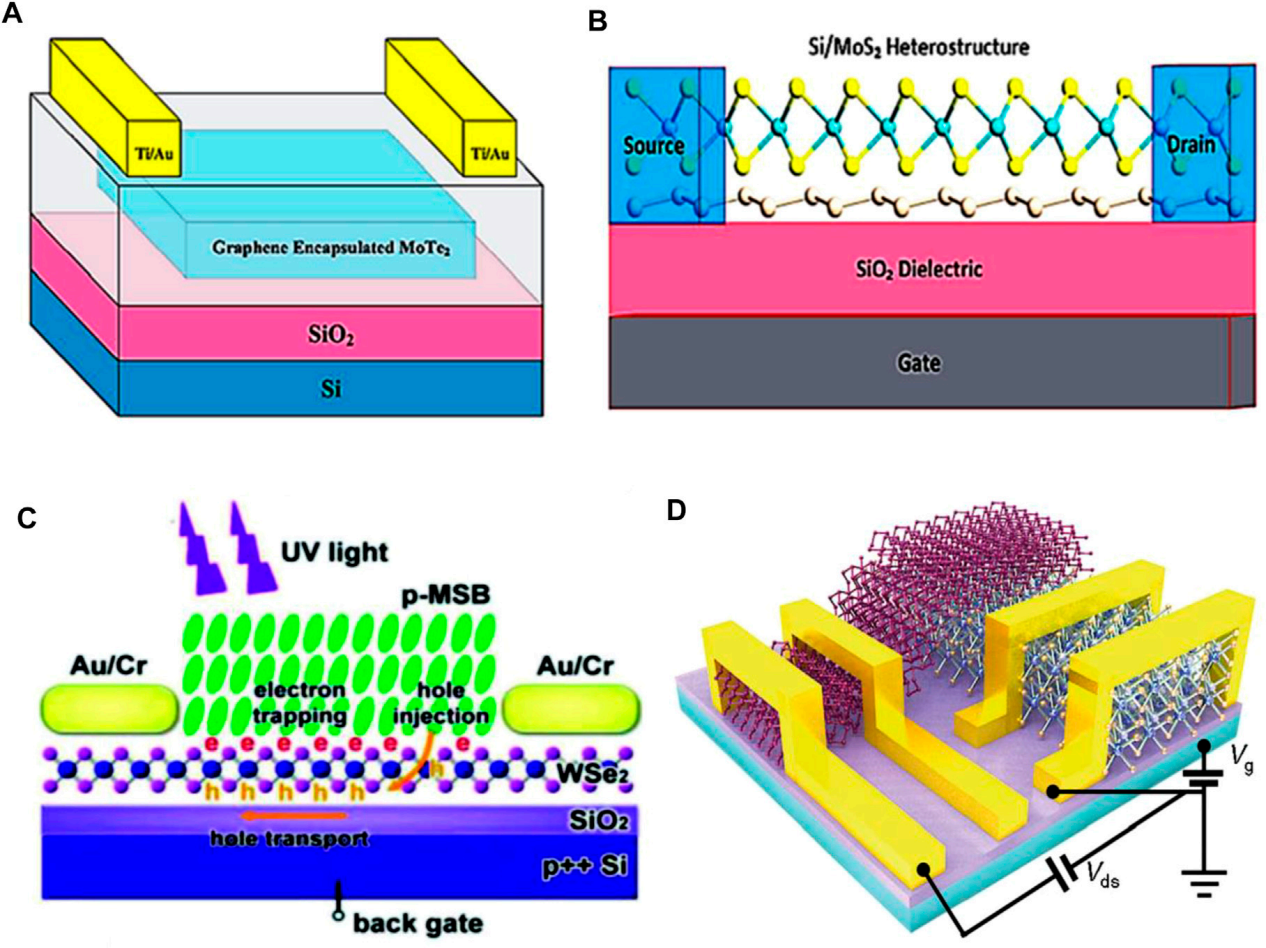
Schematic of **(A)** MoTe_2_/graphene-based photodetector (Kharadi et al., 2021), **(B)** Silicene/MoS_2_ based photodetectors (Kharadi et al., 2021), **(C)** (MSB)/WeS_2_ based photodetectors (Qiu and Huang, 2021), and **(D)** BP- WSe_2_ photodetector (Zong et al., 2020); Reproduced with permission from (Kharadi et al., 2021; Zong et al., 2020; Qiu and Huang, 2021).

Two-dimensional heterostructures are notable building blocks of photo devices in the up-to-date electronic industry. Xue et al. synthesized a MoS_2_/WS_2_ heterostructure photodetector device with a measured responsivity of 2.3 AW^−1^ at an operating wavelength of 450 nm (Xue et al., 2016). However, transfer of the synthesized heterostructure device onto the flexible substrate polydimethylsiloxane (PMDS) induces a decrease in photocurrent attributed to the trapped sites between the substrate and the active material. Cheng et al. showed that quantum efficiency of the WSe_2_/MoS_2_ heterojunction device synthesized by PVD method is high at 514 nm of operating wavelength (Cheng et al., 2014). This means that such heterojunction device is much more efficient than the WSe_2_ p-n junction (Pospischil et al., 2014). Yu et al. investigated a MoTe_2_/graphene heterostructure and demonstrated its better performance than individual material-based devices. MoTe_2_ is a photoactive material as is well known, whereas graphene provides a path for the carrier excitation (Yu et al., 2017). A phenomenon of photogating is observed in this device, which traps electrons at MoTe_2_ while holes are accumulated at graphene. Then, holes can be readily extracted owing to its high charge mobility and this enhances the performance of the device as shown in Figure 11A (Kharadi et al., 2021)**.**

Silicene, known as the “silicon version of graphene”, has many properties that resemble graphene, and has gathered great attention in the electronic industry due to its exciting features. This material can be integrated through Si-based technology. Kharadi et al. investigated a silicene/MoS_2_ heterostructure device and demonstrated that this exhibits efficient high speed carrier excitation at an operating wavelength of 650 nm. They also showed that the responsivity and detectivity of this device are 5.66 × 10^5^ AW^−1^ and 4.76 × 10^10^ Jones, respectively, as schematically described in Figure 11B (Kharadi et al., 2021). Table 1 summarizes various 2D materials and their device efficiencies. Cai et al. (Cai et al., 2018) reported the p-type 4-methylstyryl benzene (MSB)/WeS_2_ based heterostructure device. They equipped an electric tunable gating at the interface of the device in order to modulate the interfacial charges by gate voltage. This disposal enhances the device responsivity, where the resultant responsivity is 3.6 × 10^6^ AW^−1^ while detectivity is 8.6 × 10^14^ jones at an operating wavelength of 365 nm (see Figure 11C (Qiu and Huang, 2021)). On the other hand, Zong et al. reported 2D BP-WSe_2_ photodetector in order to achieve efficient electron transfer for efficient device performance. The schematic of the device is illustrated in Figure 11D (Zong et al., 2020).

**TABLE 1:** Performance yield of photodetectors based on different 2D materials.

Device type	Photoresponsivity	Photo-detectivity	Photo-response time	Operational Wavelength	Rise time/fall time
Monolayer MoS_2_ Photodetector (Yin et al., 2012)	7.5 mAW^−1^	----	50 ms	670 nm	----
WSe_2_/Graphene/MoS_2_ photodetector (Pham et al., 2022)	10^4^ AW^−1^	----	----	0.94 µm	53.6 µs/30.3 µs
Graphene-ZnO photodetector (Pham et al., 2022)	----	3.9 × 10^13^ jones	----	0.39 µm	----
Graphene-GaN photodetector (Pham et al., 2022)	10^–3^ AW^−1^	----	----	0.35 µm	----
Graphene- WS_2_- Graphene (Pham et al., 2022)	3.5 AW^-1^	10^11^ jones	----	0.532 µm	----
hBN- Graphene- WS_2_- Graphene- hBN (Pham et al., 2022)	----	----	----	0.759 µm	Rise time; 5.5 ps
Ultra-Sensitive Monolayer MoS_2_ Photodetector (Lopez-Sanchez et al., 2013)	880 AW^−1^	----	0.6 s	561 nm	4 s/9 s
Ultrasensitive multilayered MoSe_2_ back-gated FET device (Abderrahmane et al., 2014)	97.1 AW^−1^	----	15 ms	532 nm	15 ms/30 ms
MoS_2_ monolayer phototransistor by CVD fabrication method (Zhang et al., 2013a)	2.2 × 10^3^ AW^−1^	----	----	532 nm	----
Graphene—MoS_2_ (Pham et al., 2022)	1.26 AW^-1^	4.2 × 10^10^ jones	----	1.44 µm	----
MoTe_2_ –Graphene based Vis-IR photodetectors (Pham et al., 2022)	970.82 AW^-1^	1.5 × 10^11^ jones	----	1.064 µm	Rise time; 78 ms
Few-Layer WS_2_ Phototransistor (Perea ‐ López et al., 2013)	92 μAW^−1^	----	5 ms	457–647 nm	----
Graphene- ZnO photodetectors (Pham et al., 2022)	3 × 10^4^ AW^−1^	4.3 × 10^14^ jones	----	0.365 µm	----
WSe_2_ Monolayer Phototransistor (Zhang et al., 2014b)	1.8 × 10^5^ AW^−1^	10^14^ jones	Less than 23 ms	650 nm	----
ReSe_2_ nanosheet transistor (Yang et al., 2014)	95 AW^−1^	----	----	633 nm	∼68 ms/34 ms
Silicene/MoS_2_ heterostructure (Kharadi et al., 2021)	5.66 × 10^5^ AW^−1^	4.7 × 10^10^ jones	----	650 nm	----
Graphene/Carbon nanotube photodetectors (Pham et al., 2022)	100 AW^-1^	----	----	0.4–1.55 µm	Rise time; 10^–4^ s
Graphene/ZnO nanorods (Pham et al., 2022)	3 × 10^5^ AW^−1^	----	----	0.365 µm	----
GaSe- MoS_2_ UV self-driven photodetectors (Pham et al., 2022)	0.9 AW^-1^	----	5 ms	0.45 µm	----
Graphene single-wall nanotubes (Pham et al., 2022)	100 AW^-1^	----	----	0.65 µm	----
Graphene—MoS_2_ photodetector (Pham et al., 2022)	> 10^7^ AW^−1^	----	----	----	----
Graphene—InSe photodetector (Pham et al., 2022)	10^5^ AW^−1^	----	----	0.633 µm	----
ReS_2_-Graphene photodetectors (Pham et al., 2022)	7 × 10^5^ AW^−1^	1.9 × 10^13^ jones	----	0.55 µm	Rise time; <30 ms
WS_2_-PbS Vis NIR photodetectors (Pham et al., 2022)	14 AW^-1^	----	----	0.808 µm	153 µs/226 µs
MoS_2_ -PbS photodetectors (Pham et al., 2022)	5.4 × 10^4^ AW^−1^	10^11^ jones	----	0.85 µm	Rise time; 950 µs
Mono-multi MoS_2_ heterojunction Vis-NIR photodetectors (Pham et al., 2022)	1.65 × 10^4^ AW^−1^	----	----	1.064 µm	1.5 ms/2.5 ms
In- Graphene- WS_2_- graphene Vis-NIR photodetectors (Pham et al., 2022)	2.6 × 10^3^ AW^−1^	2.2 × 10^12^ jones	----	0.52 µm	40 µs/65 µs
GaSe- WS_2_ UV-Vis photodetector (Pham et al., 2022)	∼149 AW^-1^	----	----	0.41 µm	37 µs/43 µs
WSe_2_- SnSe_2_ Vis-IR photodetectors (Pham et al., 2022)	80 AW^-1^	1.4 × 10^10^ jones	----	1.55 µm	----
ZnS- MoS_2_ UV-Vis-IR photodetectors (Pham et al., 2022)	9.4 × 10^–6^ AW^−1^	----	----	0.365 µm	Rise time; 22 s
PtSe_2_- GaN UV photodetectors (Pham et al., 2022)	0.193 AW^-1^	10^14^ jones	----	0.265 µm	45 µs/102 µs

**FIGURE 12:**
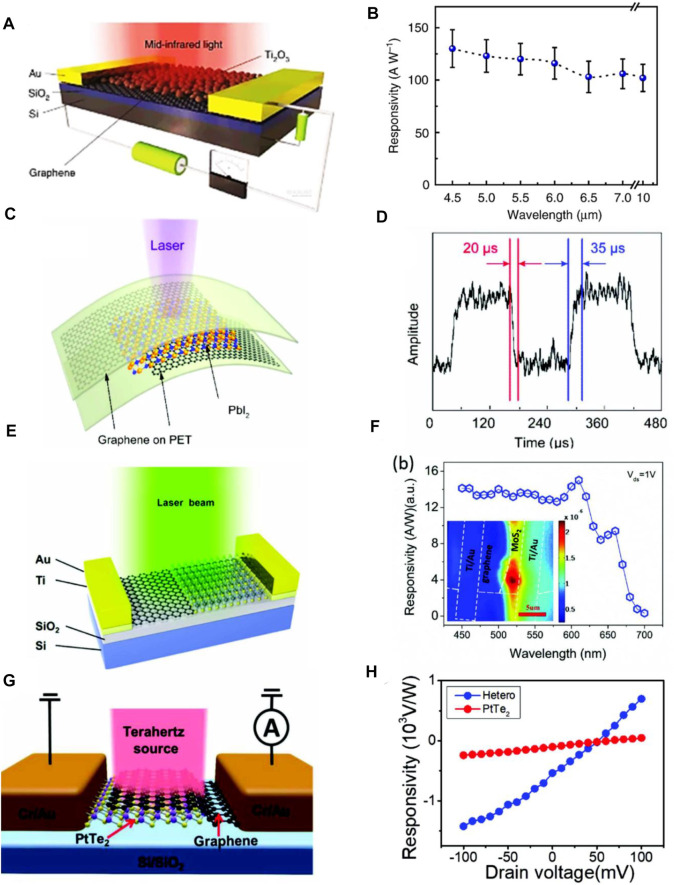
2D heterostructure photodetectors **(A**,**B)** Schematic and responsivity spectra of hybrid graphene/Ti_2_O_3_ photodetector (Yu et al., 2018a), **(C**,**D)** PbI_2_ based photodetector and its photo-response spectra (Zhang et al., 2018), **(E**,**F)** Graphene-MoS_2_ photodetector and its photoresponsivity (Deng et al., 2018b), **(G**,**H)** Schematic of PtTe_2_ based photodetector and its responsivity spectra (Xu et al., 2019); Reproduced with permission from (Yu et al., 2018a; Deng et al., 2018b; Zhang et al., 2018; Xu et al., 2019).

Various 2D material-based heterostructure photodetectors can be synthesized on conductive substrates as well as on flexible ones with enhanced photo-responsivity and short response times (Zong et al., 2020; Yu et al., 2018a; Zhang et al., 2018; Deng et al., 2018b; Xu et al., 2019). Yu et al. investigated graphene/Ti_2_O_3_ photodetectors with a responsivity and detectivity of 300 AW^−1^ and 7×10^8^ jones, respectively, wherein a high responsivity was observed at 4.5–10 μm wavelength, depicting the wide spectral band function of the hybrid graphene/Ti_2_O_3_ photodetectors (Figures 12A,B) (Yu et al., 2018a). However, the error bars in Figure 12B illustrate the standard deviation. It was reported that PbI_2_/graphene-based photodetectors are best for the application of efficient photodetectors, since the capability of their light absorption is high, whereas they exhibit a short response time as shown in Figures 12C,D (Zhang et al., 2018). Enhancing light detectivity of detectors as well as fabrication of stabled devices are the recent challenges that the optoelectronic industry is facing for their commercial applications. To address these challenges, Deng et al. made a graphene-MoS_2_ heterostructure photodetector and reported that its absorptivity power and detectivity are high compared to others. They also proved that photogenerated charge carrier separation, in that device, is efficient and charge transportability is high, whereas its photo-responsivity is 10^5^ AW^−1^. Regarding this, the schematic of the device and photo-responsivity spectra is demonstrated in Figures 12E,F (Deng et al., 2018b). Furthermore, Xu et al. studied PtTe_2_-graphene heterostructure photodetector and they showed that its photo-responsivity is good in the absence of an external electric field. They also explored applicability of that device in high-performance photodetection. The schematics of this device together with its photo-responsivity spectra are shown in Figures 12G,H (Xu et al., 2019).

## 6 Photodetection Wavelength Ranges

### 6.1 Photodetectors in UV Region

Ultraviolet wavelength-based devices are widely used thanks to their applicability in rich technologies from large-scale (space science) to atomic-scale (photodetection). The conventional UV-range photodetectors based on various active materials show a low responsivity of 0.1–0.2 mAW^−1^ (Hochedez et al., 2009). Such poor device efficiency is caused by a lattice mismatch between the active material and the substrate (Kong et al., 2016). Besides, settling of some imminent problems, such as the cost and complex configuration of devices together with the mismatch between the bandgap of the active material and illuminated light energy, could be a challenge in devising and fabricating up-to-date photodetectors. This is the reason why skillful strategies are necessary in the design of 2D materials-based UV detectors.

**FIGURE 13:**
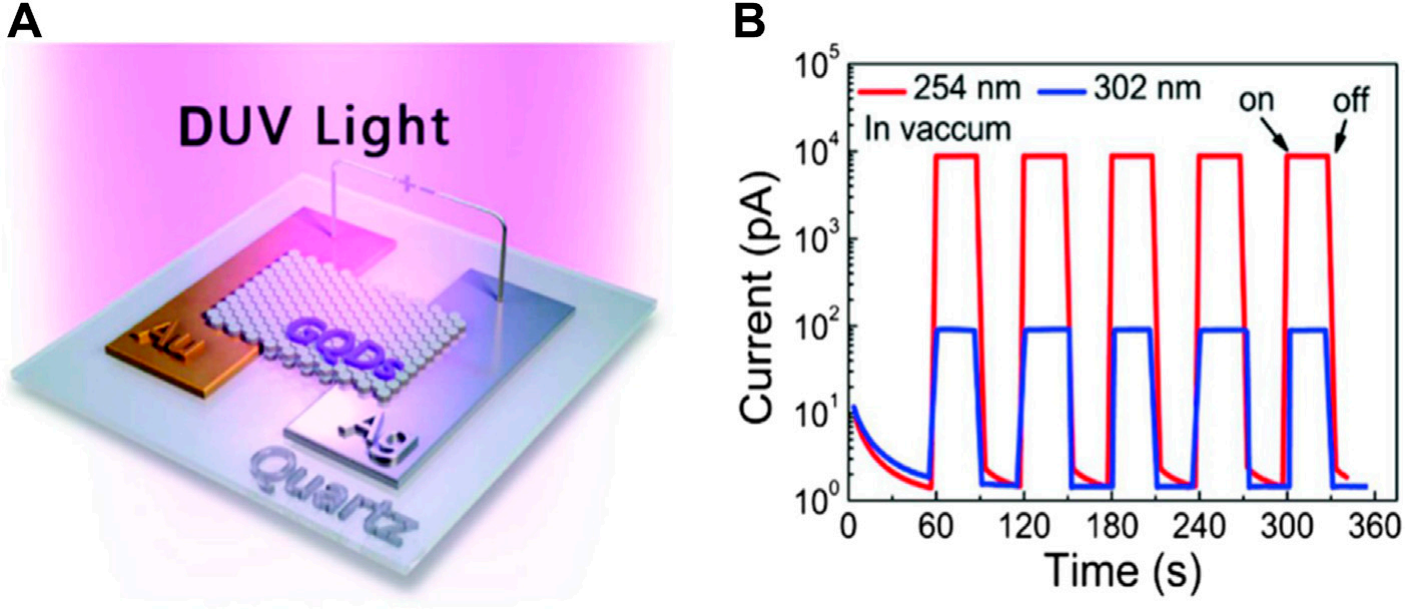
**(A)** Schematic of graphene-QD-based photodetector (Zhang et al., 2015), **(B)** Time response of graphene-QD-based photodetector (Zhang et al., 2015); Reproduced with permission from (Zhang et al., 2015).

The first strategy along this line may be minimizing the illuminated light reflection and absorption by replacing metal electrodes with graphene. A Schottky UV detector based on graphene/GaN shows the advantage of transparency under UV illumination (Xu et al., 2015). Such a transparency allows more light to fall on the GaN active material, leading to enhancing the device’s light absorptivity. Owing to graphene’s tunable bandgap, doping levels in graphene can augment the inherent electric field. The second strategy is to combine the wide bandgap semiconductors with graphene in order to ensure UV range photodetection. In a recent report, ZnO nanostructures having a large direct bandgap which is 3.37 eV together with a high exciton binding energy of 60 meV were combined with graphene for the purpose of synthesizing a hybrid structure photodetector (Soci et al., 2007). Furthermore, ZnO nanostructures, such as nanoparticles, nanowires, nanoribbons, and quantum dots, can be combined with graphene to create hybrid photodetectors. Such nanostructures are more sensitive in photon detection than conventional ZnO-based photodetectors. In this class of combined detectors, active material and graphene are joined by a Schottky junction (Fu et al., 2012; Dang et al., 2015). The third strategy is a practical application of bandgap engineering. That is, this strategy is an enhancement of the UV illumination sensitivity by modifying the graphene bandgap in a way that its size being 0D quantum dots. This improvement may result in a UV-matched modulated bandgap owing to graphene’s quantum confinement effects (Yan et al., 2010). Zhang et al. used graphene quantum dots in the UV range photodetection for the purpose of modifying light sensitivity. This device exhibits a high on/off ratio of 6000 at a quite weak intensity of incident light. Such a notable result is attributed to the minimized carrier transportation barriers, as schematically shown in Figure 13A (Zhang et al., 2015). The time response of graphene-QD based photodetector is depicted in Figure 13B (Zhang et al., 2015)

Black phosphorus is also reported as an effective material in the application of UV ranged photodetection. For instance, due to the inter-band transition between the empty valance and conduction bands, BP shows a high device responsivity of 90000 AW^−1^. This means that BP-based photodetectors are much more efficient than conventional UV ranged photodetectors.

**FIGURE 14:**
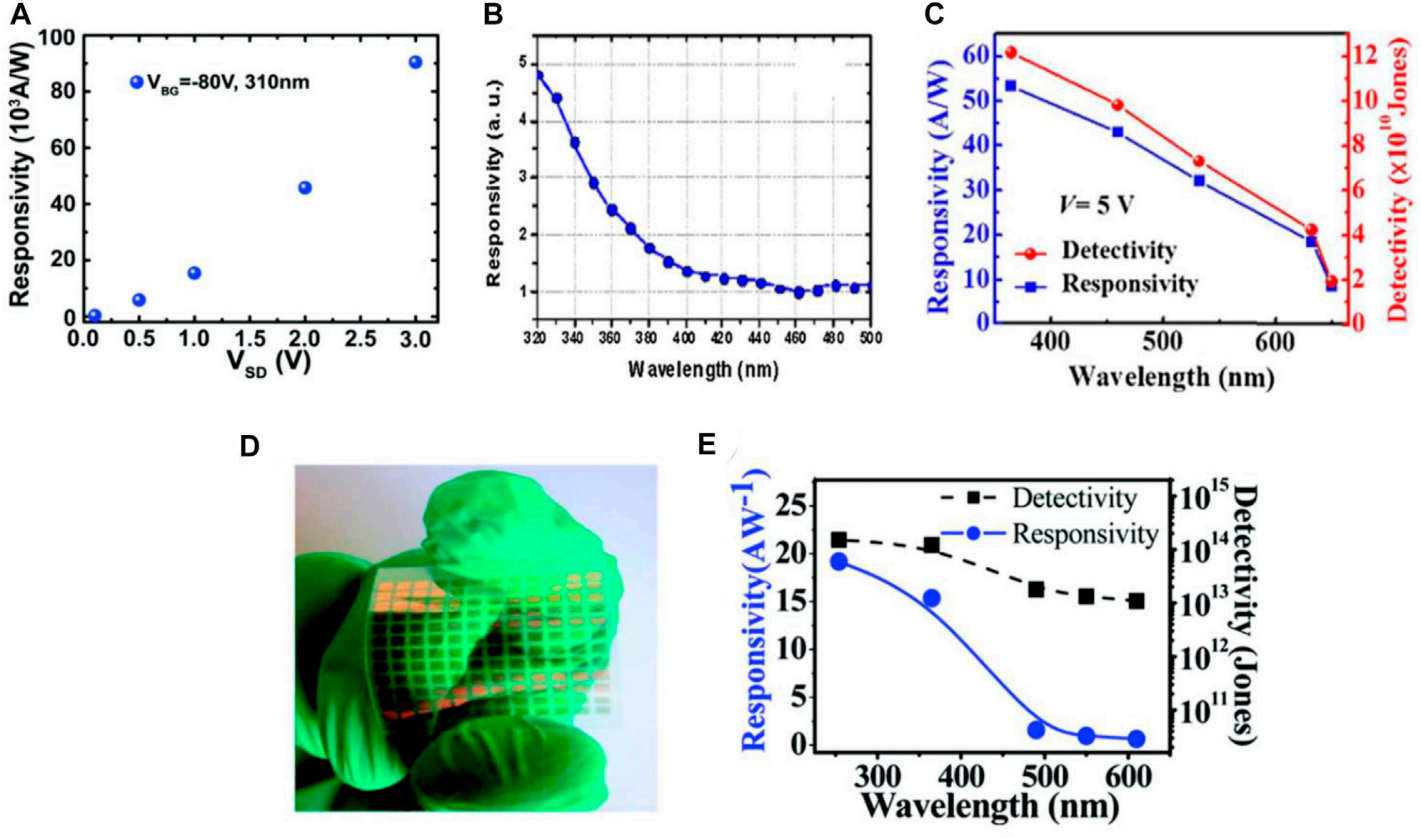
**(A)** Photoresponsivity of BP based photodetector (Wu et al., 2015), **(B)** Photoresponsivity of MoS_2_-NCs based photodetector (Alkis et al., 2012), **(C)** Photoresponsivity and detectivity of WS_2_ based photodetector (Zeng et al., 2016), **(D)** Optical image of GaS thin film-based photodetector (Hu et al., 2013), and **(E)** Responsivity and photo-detectivity of GaS thin film-based photodetector (Hu et al., 2013); Reproduced with permission from (Hu et al., 2013; Wu et al., 2015; Alkis et al., 2012; Zeng et al., 2016).

Alkis et al. reported a MoS_2_ nanocrystal-based photodetector applicable to UV photodetection within the wavelength range of 300–400 nm (Alkis et al., 2012). Photo-responsivity of BP is shown in Figure 14A (Wu et al., 2015), while Figure 14B (Alkis et al., 2012) depicts responsivity for a MoS_2_-NCs based photodetector. Zeng et al. synthesized WS_2_-based photodetectors and showed that they exhibit high responsivity and detectivity of 53.3 AW^-1^ and 1.22 × 10^11^ jones, respectively. There excellent photo-responsivity and detectivity result is shown in the Figure 14C (Zeng et al., 2016b). Another promising scheme for photodetection along this line is the use of photodetectors fabricated based on III–V materials. For instance, GaS emits blue light owing to its direct and indirect bandgaps at 2.59 and 3.05 eV, respectively. Hu et al. investigated GaS thin film-based photodetectors fabricated on a flexible polymer substrate. They reported that the responsivity and detectivity of their fabricated devices are 19.2 AW^−1^ and 10^14^ jones, respectively [see Figures 14D,E (Hu et al., 2013)]. Based on these outcomes, we can conclude that the devices exhibit very high quantum yield compared to traditional graphene photodetectors.

### 6.2 Photodetectors in Near-IR Region

**FIGURE 15:**
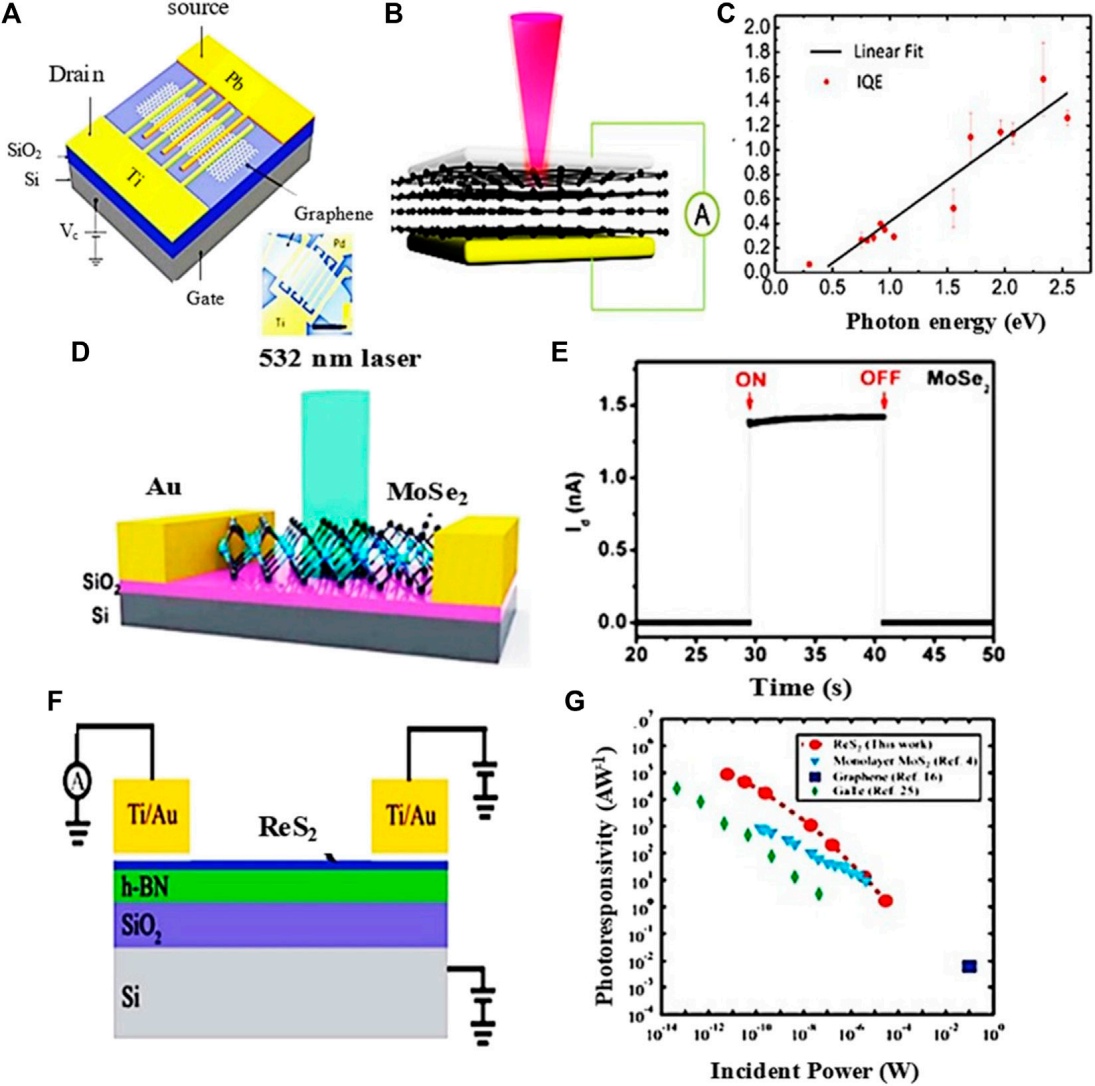
**(A)** Graphene photodetector with different metal contacts (Mueller et al., 2010), **(B)** Schematic of graphene stacked device (Chen et al., 2015), **(C)** Graphene stacked based photon energy dependence on IQE, **(D)** Monolayered MoSe_2_ photodetector (Wang et al., 2018), **(E)** With its time resolved current (Wang et al., 2018), **(F)** Multilayered RSe_2_ photodetector (Liu et al., 2016b), and **(G)** With its responsivity (Liu et al., 2016b); Reproduced with permission from (Mueller et al., 2010; Liu et al., 2016b; Chen et al., 2015; Wang et al., 2018).

The visible and near-IR range photodetectors are widely studied because of their actual importance of application in science and technology. The performance of photodetectors depends on their energy level whereas easy and efficient separability of charge carriers is necessary. Graphene-based photodetectors with different metal electrodes can be used for source and drain fabrications. In these devices, charge carriers can be efficiently separated by incident light, where the corresponding highest responsivity obtained is 6.1 mAW^−1^ (see Figure 15A (Mueller et al., 2010)). Chen et al. reported different metal contact-based vertically-stacked layer graphene devices operating in the visible to mid-IR wavelength range. The detection mechanism underlying these devices is directly related to the carrier multiplication effect (see Figures 15B,C) (Chen et al., 2015).

A drawback of graphene is its zero-bandgap. Apart from graphene, 2D TMDC materials including MoS_2_, MoSe_2_, WS_2_, WSe_2_, RS_2_, and RSe_2_ can be effectively used for the purpose of photodetection (Mukherjee et al., 2015; Zhang et al., 2016c). MoS_2_ has an indirect bandgap of 1.2 eV in bulk and this can be modified to 1.8 eV by making the material in the form of a nanosheet (thin film). This material is suitable for NIR ranged detection with a low dark current and high on/off ratio, since it matches well with the incident NIR wavelength illumination (Lopez-Sanchez et al., 2013; Radisavljevic et al., 2011). The single-layered MoS_2_ based photodetectors exhibit the responsivity of 880 AW^−1^ at an operating wavelength of 561 nm (Lopez-Sanchez et al., 2013). MoSe_2_ can be favorably used in spintronics owing to its high spin splitting energy. The response time of this material is relatively high compared to the MoS_2_ devices (Figures 15D,E) (Wang et al., 2018). Liu et al. fabricated a multilayered ReS_2_-based photodetector and showed that its response at an operating wavelength of 532 nm is 88600 AW^−1^ (Figures 15F,G) (Liu et al., 2016b). This response is efficient compared to that of the single-layered RSe_2_-based device.

**FIGURE 16:**
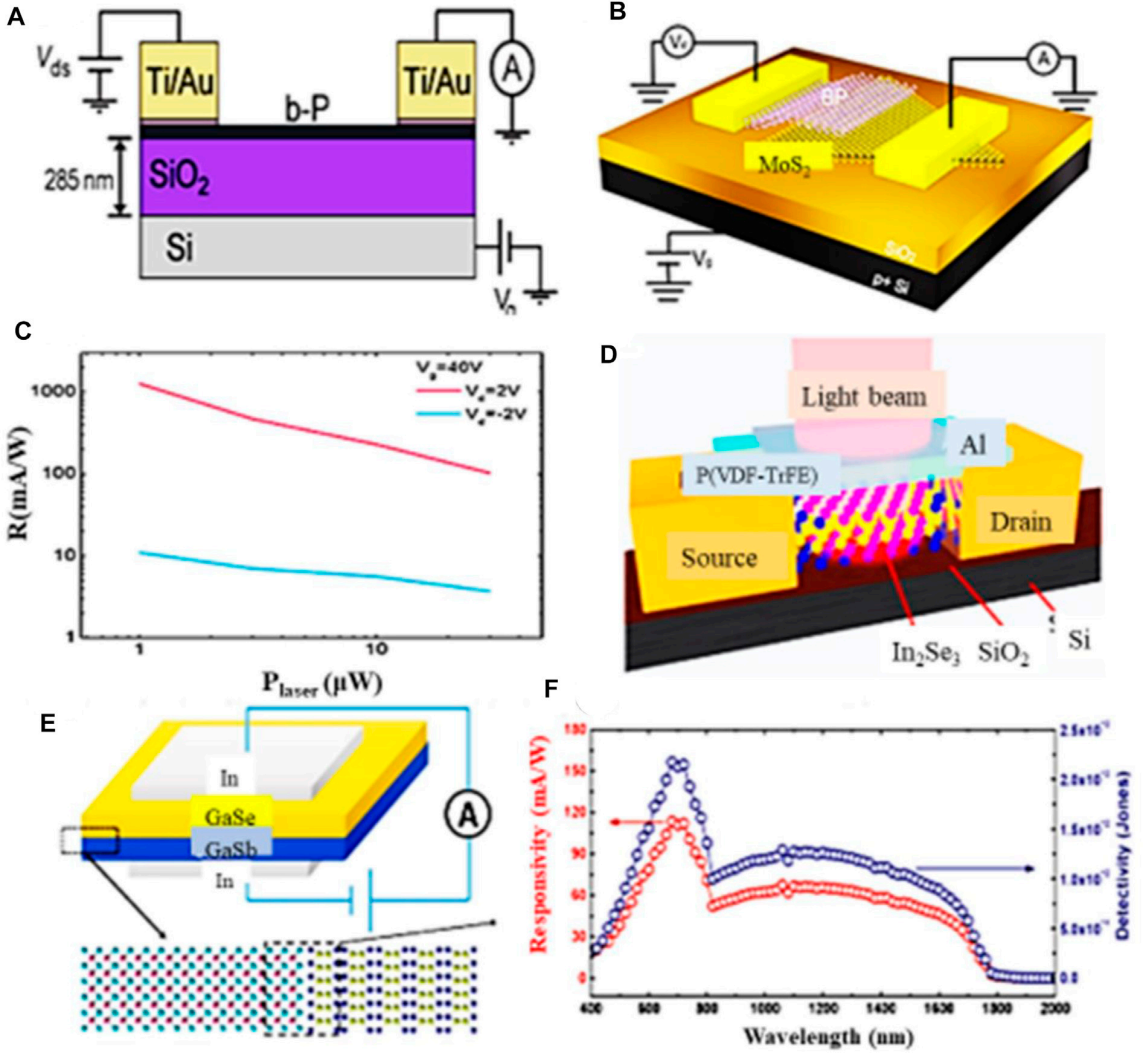
**(A)** schematic of BP based photodetector (Buscema et al., 2014), **(B)** schematic of BP-MoS_2_ based photodetector (Deng et al., 2014), **(C)** BP-MoS_2_ based photodetector responsivity, **(D)** schematic of InSe based photodetector (Wu et al., 2016), **(E)** schematic of GaSe-GaSb based heterostructure photodetector (Wang et al., 2017), and **(F)** GaSe-GaSb based heterostructure photodetector responsivity (Wang et al., 2017); Reproduced with permission from (Buscema et al., 2014; Deng et al., 2014; Wu et al., 2016; Wang et al., 2017).

Black phosphorus is also a good material for the photodetection thanks to its wide bandgap (0.3–1.9 eV) that makes the dark current small compared to graphene. The reason that makes BP a best suit for photodetection applications is that it has high charge carrier mobility even at room temperature in contrast with 2D TMDCs (Xia et al., 2014b). Buscema et al. reported a high response speed BP-based photodetector with a rise and fall time of about 1 and 4 ms, respectively, at an operating wavelength of 940 nm (Figure 16A) (Buscema et al., 2014). In another study, Li et al. analyzed WSe_2_/BP/MoS_2_ heterostructure phototransistor presenting its broadband photoresponse from visible to the infrared spectral regions, with photo-responsivity values of 6.32 and 1.12 AW^‐1^, respectively, which are both improved by tens of times in comparison with similar photodiodes composed of WSe_2_-BP, indicating its unique merit of photocurrent amplification capacity (Li et al., 2017). Deng et al. demonstrated that gate tunable current in BP-MoS_2_ heterostructure device can be rectified with a high responsivity of 418 mAW^−1^ (Deng et al., 2014; Deng et al., 2021), which is 100 times more efficient than intrinsic BP photodetectors and 26 times more efficient than previously studied WSe_2_ based photodetectors (Figures 16B,C) (Deng et al., 2014).

Aside from these, III–V materials can be suitably adapted in NIR photodetection. As shown in Figure 16D (Wu et al., 2016), InSe-based photodetectors fabricated on a ferroelectric material exhibit a fast response time of 200 s at an illumination wavelength of 1550 nm and the dark current observed in these detectors is low due to the large intrinsic electric field of their ferroelectric material. Besides, other heterostructure photodetectors were also investigated. For example, Wang et al. reported GaSe-GaSb based photodetectors applicable in detection with a wide wavelength range of 400–1800 nm. They showed that these detectors exhibit an enhanced response time of 20–30 µs along with Vis-NIR detectivites of 2.2 × 10^12^ and 1.3 × 10^12^ jones, respectively (Figures 16E,F) (Wang et al., 2017). A strong electrical field is attributed to the minimized dark current and charge carrier separation, which is the key parameter for wide-band detection with enhanced photo-response time.

### 6.3 Photodetectors in Mid and Far IR Region

**FIGURE 17:**
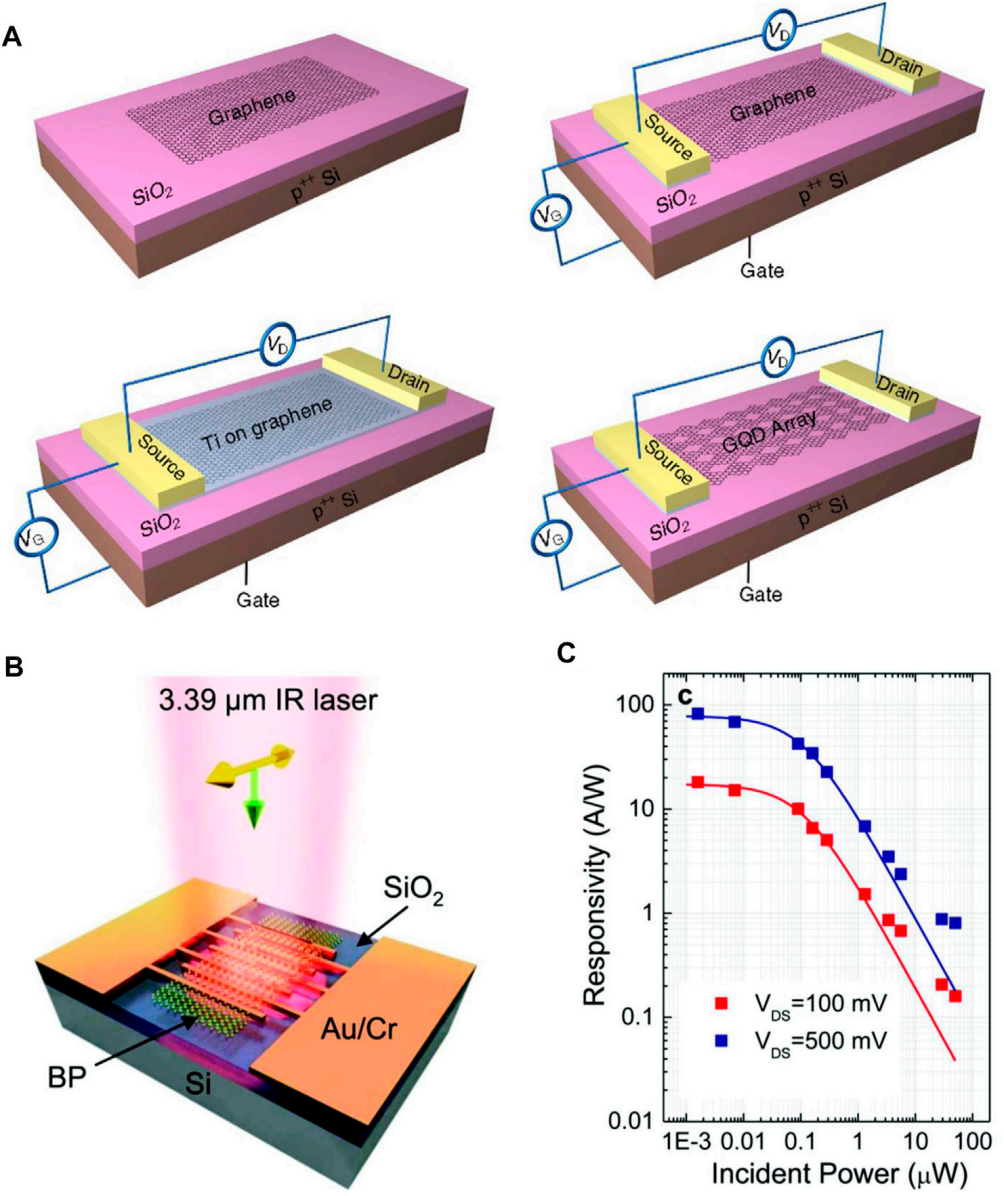
**(A)** Fabrication process of graphene-based photodetector band engineering (Zhang et al., 2013b), **(B)** Schematic of BP based photodetectors, and **(C)** BP based photodetector responsivity (Guo et al., 2016); Reproduced with permission from (Zhang et al., 2013b; Guo et al., 2016).

Mid and far-IR ranged photodetectors have broad technological applications. The drawbacks of conventionally reported HgCdTe IR photodetector are its complexity and relatively high fabrication cost. For the case of quantum well IR photodetectors, they require low operation temperature and exhibit high dark current, which limit their applications (Sudradjat et al., 2012; Hofstetter et al., 2003). To overcome these problems of existing detectors, 2D materials that have interesting features in both near and far IR detections emerge as alternatives. Detection wavelengths from MIR to THz can be covered by graphene owing to its weak electron-phonon coupling and small electron heat capacity. While graphene is suitable for photo-thermal detectors, the detections for MIR and FIR illuminations are accompanied by a large variation of temperature. Using band engineering technique for graphene-based photodetectors, it is possible to tailor their bandgap in a way that they exhibit a high photoresponsivity. Through this, Zhang et al. obtained responsivity of 0.4 AW^−1^ for a graphene-based photodetector at an operating wavelength of 10 µm as illustrated in Figure 17A (Zhang et al., 2013b). Reduced-graphene oxide (rGO) having a high bandgap was also reported. The resistance of rGO decreases as a function of light-illumination time, whereas the generated photocurrent is localized in defect sites and residual oxygen groups. In the illumination of light of which photon energy is smaller than rGO bandgap energy, photodetection can be attained through thermal excitation of electrons typically (Liang, 2014).

In NIR to MIR detections, black phosphorus can also be potentially used depending on the material thickness and light polarization effects (Low et al., 2014). A polarization-sensitive p-n junction MIR photodetector is analyzed with an operating wavelength of 3000–3750 nm (Yuan et al., 2015). Though different light polarizations in different directions were observed through this, it exhibits an efficient response through a broad-spectrum range in the armchair direction. Guo et al. investigated BP-based photodetectors having a photoresponsivity of 82 AW^−1^ at an illumination wavelength of 3.39 µm and a sensibility of pW range. We have represented the demonstration of such outcomes in Figures 17B,C (Guo et al., 2016).

Owing to the dynamics of quick carriers in relation with BP’s moderate bandgap, the fast photo-response works successfully at kilohertz (KHz) modulation frequencies. Suess et al. investigated an ultrasensitive BP-based MIR photodetector which exhibits a fast response speed at room temperature. They demonstrated that the rise time of this device is 65 ps and is relatively fast, whereas its NEP is 530 pWHz^1/2^ (Suess et al., 2016). We can confirm by regarding this outcome that BP is an efficient material for MIR optoelectronics. Indeed, BP exhibits a high responsivity together with a rapid and sensitive photo-response.

The capacity of commercially available photodetectors for FIR detection is mostly reliant on thermal response. This necessitates cryogenic cooling for the operation of such systems in order to minimize the dark current. Graphene, on the other hand, is frequently employed as an auxiliary material in research for increasing the performance of traditional FIR photodetectors. It is reported that the graphene-GaAs/AlGaAs composite-based quantum-Hall-states FIR photodetector has a good responsivity due to its great carrier density of 2D electron gas (Tang et al., 2014).

## 7 Perspectives and Proposed Factors for Device Improvement

The responsivity, detectivity, response time, and NEP are the key parameters used for estimating the quality in the performance of photodetectors. The efficiency in photodetection relies on the improvement of certain parameters, such as enhancement of the light absorption power, minimization of the dark current, and widening of the response range.

### 7.1 Defect Engineering

Modification of the bandgap of targeted materials can be carried out through the defect engineering, also known as the bandgap engineering, which eventually enhances the device performance at the expense of the increased light response time. That is, this procedure limits the response time of the device. Xie et al. modulated the MoS_2_ material by introducing Mo^4+^ and S_2_ vacancies through the defect engineering (Xie et al., 2020). Based on this technique, they improved the device performance by achieving a smaller bandgap which entails high carrier concentrations. A similar modification for bilayer PtSe_2_ on the basis of defect engineering was also reported by Yu et al. (Yu et al., 2018b). They achieved 4.5 AW^−1^ of responsivity and 7 × 10^8^ jones of detectivity at an operating wavelength of 10 µm for that material. Evidently, defect engineering could be a possible solution for enhancing device performance associated with the material’s bandgap, in which materials thickness induces more defects and these defects, consequently leads to tailor the bandgap in a specific range (Zhong et al., 2021).

### 7.2 Interface and Electrode Engineering

Popular knowledge predicts that surface band bending and interface decomposition are substantial impediments to reaching the contact energy alignment that popular knowledge predicts. Contact parameters can differ between findings from various laboratories due to differences in perovskite synthesis and interfacial reactions. Given these challenges, contact engineering to remove or at the very least decrease parasitic interfacial effects is critical for perovskite electronics to go further. In 2D perovskite-based optoelectronic devices, interface and electrode engineering are being used to achieve ohmic transport and minimize hysteresis (Lin et al., 2022).

### 7.3 Hybrid Detectors

Hybrid devices are the combinations of various materials, including organic materials, ferroelectric materials, and nanoparticles. These may exhibit diverse advantages for a wide response range caused from the combining of different materials. The device responsivity of photodetectors can be improved by graphene-based hybridization in their fabrications. For instance, responsivity of three graphene-based photodetectors made by hybridization of ZnS, ZnSe, and CdSe nanosheets were reported as 1.7 × 10^7^, 3.9 × 10^6^, and 2.3 × 10^6^ AW^−1^, respectively (Huang et al., 2016). From these measured values, we can confirm actual enhancement of the responsivity. Graphene deposition over Si was also reported for a high frequency-range (GHz to THz) hybrid photodetector with a responsivity of 2 × 10^4^ AW^−1^ at room temperature by Amirmazlaghani et al. (Amirmazlaghani and Raissi, 2018). They showed that the dominant device mechanism behind such a resultant responsivity is the photo-thermoelectric effect.

### 7.4 Nanostructures

The use of nanostructured devices could also be a suitable solution to enhance photodetector performance. Nanostructures in this context can exploit the enhanced carrier multi-excitation. Such excitation occurs owing mainly to the exposition of the material to high-energy photons. This may compensate excitations originated from low-energy photons. Hence, it is possible to attain high-response photoelectric detection without compromising the ultrafast broadband operation. Cakmakyapan et al. used the gold-patched graphene nanostructures in order to achieve a fast photodetection through a wide frequency range (Cakmakyapan et al., 2018). They reported that the responsivity of that device is 0.6 AW^−1^ and 11.5 AW^−1^ at operating wavelengths of 0.8 and 20 µm, respectively.

### 7.5 Substrates

The substrate also plays a critical role in device performance. The limitation of the light absorption power for the active material, which strongly affects photodetectors performance, is mainly depends on the characteristic of used substrate. Conventionally, Si and SiO_2_ based substrates are adopted in photodetector devices. Such class of substrates has considerable surface defects and maintains highly active charge-trapping capacity. These make dark current high and photo-response time increase. Hence, the use of an appropriate device substrate could be a potential way in order to make the gain in photodetectors sufficiently high.

### 7.6 Future Prospects

**FIGURE 18:**
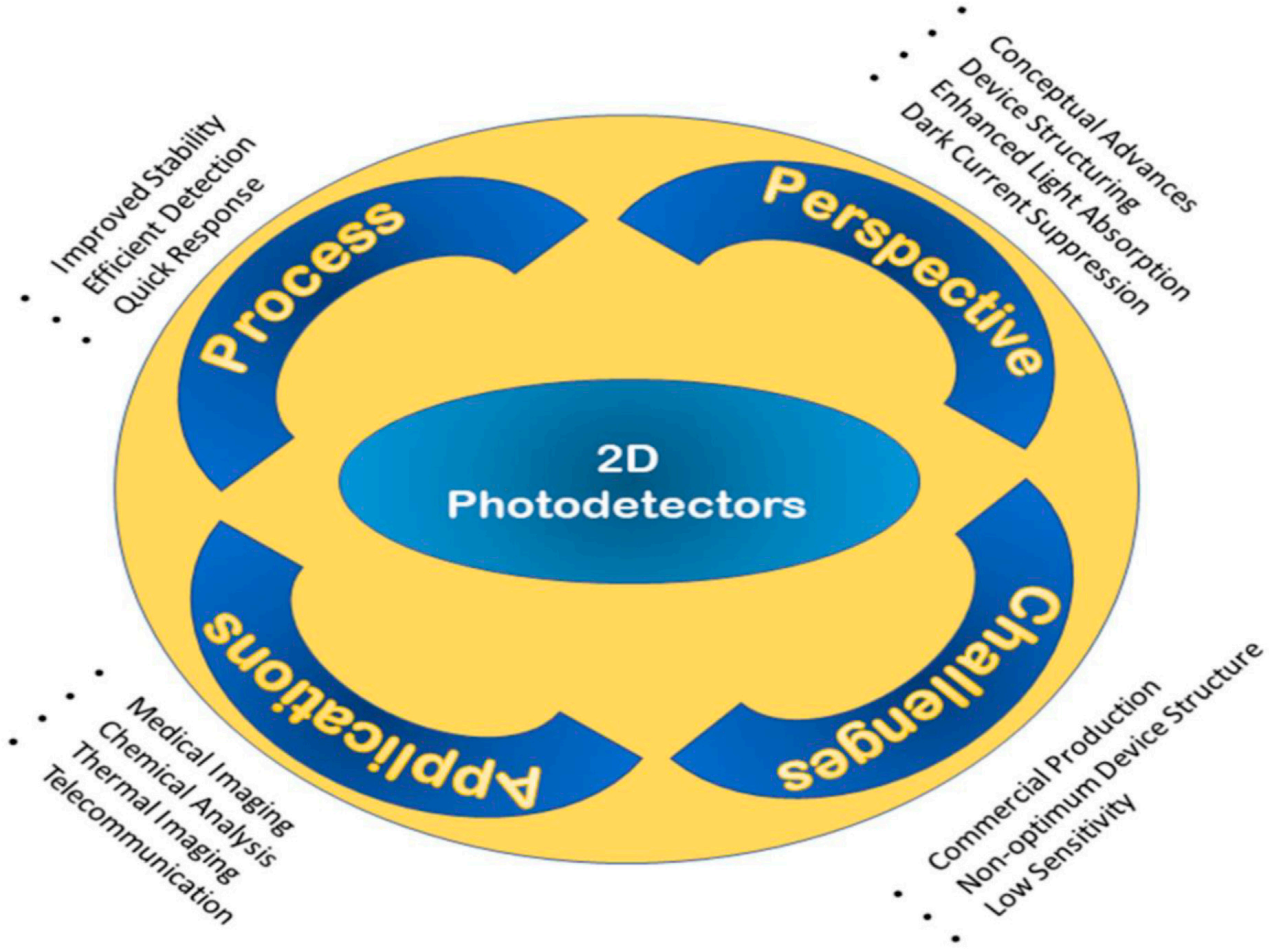
2D materials-based photodetector processes, perspectives, challenges, and applications.

To summarize, various 2D materials, which emerged over the years, can be potentially used for the purpose of photodetection due to their remarkable photonic properties. However, alongside their wide applications in medical imaging, telecommunication, as well as other sensing applications, they still face various challenges. First of all, large-scale production of state-of-the-art photodetectors is not possible yet due to the non-optimal synthesis techniques, high cost, and non-efficiency in material processes. Furthermore, the contact barrier between the 2D materials and the electrode is still relatively high, and the stability of the device is poor. More importantly, in addition to the aforementioned prospective methods for photodetector improvement, many new photodetector mechanisms need to be further explored. Some of them are hunting for new materials, improving device structure, suppressing dark current, and enhancing the material’s light absorptivity power for high performance. Though extensive research was devoted in this context so far, it may be highly necessary to develop additional high-performance 2D materials in the future. Along this, it is anticipated that photodetectors based on 2D materials will have promising development and application possibilities. Figure 18 depicts a summary of 2D materials-based photodetector processes, perspectives, challenges, and applications.

## 8 Summary and Outlook

We reviewed the performance of 2D material-based photodetectors in the detection range from UV to IR, focusing on recent progress of the past few years. Photonic features and fabrication methods for potential 2D materials are discussed. In particular, the noticeable properties of these molecules associated with their applications in photodetection, including possible strategies for synthesis, are addressed in detail. Besides, mechanisms underlying the operation of state-of-the-art 2D materials-based photodetectors, device performance parameters, and their detectivity ranges are represented. Some of the challenges, such as stabilization of devices, uplift of 2D-material compatibility on photo-detection, and the problem of electrode contact, are also outlined. The main future tasks in this context are resolving the limitations of device performance, quality enhancement by improving sensitivity, simplification of synthesizing process, cost-effective production of 2D photonic materials, and their commercialization. We expect that, by optimizing the surface of the material and engineering appropriate device architectures, 2D materials will find their way to superiority in future optoelectronic and nanophotonic devices.
